# Phylogenetic reassessment of tribe Anemoneae (Ranunculaceae): Non-monophyly of *Anemone* s.l. revealed by plastid datasets

**DOI:** 10.1371/journal.pone.0174792

**Published:** 2017-03-31

**Authors:** Nan Jiang, Zhuang Zhou, Jun-Bo Yang, Shu-Dong Zhang, Kai-Yun Guan, Yun-Hong Tan, Wen-Bin Yu

**Affiliations:** 1 Southeast Asia Biodiversity Research Institute & Center for Integrative Conservation, Xishuangbanna Tropical Botanical Garden, Chinese Academy of Sciences, Mengla, Yunnan, China; 2 Zhejiang Institute of Subtropical Crops, Zhejiang Academy of Agricultural Sciences, Wenzhou, Zhejiang, China; 3 Plant Germplasm and Genomics Center, Germplasm Bank of Wild Species, Kunming Institute of Botany, Chinese Academy of Sciences, Kunming, China; 4 Xinjiang Institute of Ecology and Geography, Chinese Academy of Sciences, Urumqi, Xinjiang, China; National Cheng Kung University, TAIWAN

## Abstract

Morphological and molecular evidence strongly supported the monophyly of tribe Anemoneae DC.; however, phylogenetic relationships among genera of this tribe have still not been fully resolved. In this study, we sampled 120 specimens representing 82 taxa of tribe Anemoneae. One nuclear ribosomal internal transcribed spacer (nrITS) and six plastid markers (*atpB-rbcL*, *matK*, *psbA-trnQ*, *rpoB-trnC*, *rbcL* and *rps16*) were amplified and sequenced. Both Maximum likelihood and Bayesian inference methods were used to reconstruct phylogenies for this tribe. Individual datasets supported all traditional genera as monophyletic, except *Anemone* and *Clematis* that were polyphyletic and paraphyletic, respectively, and revealed that the seven single-gene datasets can be split into two groups, i.e. nrITS + *atpB-rbcL* and the remaining five plastid markers. The combined nrITS + *atpB-rbcL* dataset recovered monophyly of subtribes Anemoninae (i.e. *Anemone* s.l.) and Clematidinae (including *Anemoclema*), respectively. However, the concatenated plastid dataset showed that one group of subtribes Anemoninae (*Hepatica* and *Anemone* spp. from subgenus *Anemonidium*) close to the clade *Clematis* s.l. + *Anemoclema*. Our results strongly supported a close relationship between *Anemoclema* and *Clematis* s.l., which included *Archiclematis* and *Naravelia*. Non-monophyly of *Anemone* s.l. using the plastid dataset indicates to revise as two genera, new *Anemone* s.l. (including *Pulsatilla*, *Barneoudia*, *Oreithales* and *Knowltonia*), *Hepatica* (corresponding to *Anemone* subgenus *Anemonidium*).

## Introduction

Tribe Anemoneae is a member of subfamily Ranunculoideae (Ranunculaceae) [1–4]. Traditionally, this tribe included three subtribes, i.e., Anemoninae, Clematidineae and Kingdoniae [1–3]. An overview of classifications for tribe Anemoneae is summarized in S1 Table. The subtribe Kingdoniae contains only one species, *Kingdonia uniflora* Balf. f. & W. W. Sm., which is characterized by one cordate-orbicular leaf, veins bifurcated and a short flower stalk with a small flower. *Kingdonia uniflora* grows at high elevations in western China [5]. Currently, morphological and molecular evidences show that *K*. *uniflora* should be excluded from tribe Anemoneae, even from Ranunculaceae [6], and it has been treated as an independent family Kingdoniaceae, or incorporated into family Circaeasteraceae since 2009 [4, 7]. Excluding *K*. *uniflora*, tribe Anemoneae was strongly supported as monophyletic in phylogenetic analyses [4, 8–11].

Traditionally, subtribe Clematidinae comprised three genera: *Archiclematis* (Tamura) Tamura, *Clematis* L., and *Naravelia* Adans. [1–3]. The largest genus *Clematis* has more than 300 species [12]. In some classification systems, this genus was treated as several genera on the basis of morphological, palynological, and anatomical data, e.g., *Atragene* L., *Cheiropsis* (DC.) Bercht. ex J. Presl, *Clematopsis* Bojer ex Hutch., *Meclatis* Spach, *Viorna* (Pers.) Rchb. [12]. In general, these ranks have been adopted as sections or subgenera under *Clematis* [12–16]. The flower of *Archiclematis alternata* (Kitam. & Tamura) Tamura (≡ *Clematis alternata* Kitam. & Tamura) resembles *Clematis* section *Viorna* (Reichb.) Prantl. [17], while this is the only species having alternate leaves in this subtribe. Wang and Li [12] treated *Archiclematis* as a section in *Clematis*. *Naravelia* is restricted to tropical Asia. In the full revision of *Naravelia*, Tamura [18] accepted seven species. *Naravelia* is distinguished from *Clematis* with the presence of petals and leaflet tendrils. According to molecular phylogenetic analyses [19, 20], *Clematis* is paraphyletic, including *Naravelia* and *Archiclematis*. However, the status of *Naravelia* need to be further confirmed because the studies [19, 20] included only two species without the generic type, i.e. *N*. *eichleri* Tamura and *N*. *laurifolia* Wall. ex Hook. f. & Thomson. Wang et al. [4] documented that *Naravelia zeylanica* L. is the sister to *Clematis*, though this study only included one *Clematis* species, *C*. *ganpiniana* (H. Lév. & Vaniot) Tamura.

Generally, subtribe Anemoninae consists of eight genera: *Anemoclema* (Franch.) W. T. Wang, *Anemone* L., *Barneoudia* C. Gray, *Hepatica* Miller, *Knowltonia* Salisb, *Metanemone* W. T. Wang, *Oreithales* Schldl., and *Pulsatilla* Mill. [1–3, 21, 22]. Among them, *Anemoclema*, *Metanemone* and *Oreithales* are monotypic (i.e., only one species). The genus *Anemone* contained more than 150 species, and it is distributed throughout the world. Molecular phylogenetic studies recognized that *Hepatica*, *Pulsatilla* and *Knowltonia* are nested within *Anemone*, and that they should be subsumed within *Anemone* [23–25]. Then, Hoot et al. [26] and Mayer et al. [25] revealed that two South American endemic genera *Barneoudia* and *Oreithales* should be also included in *Anemone*. *Anemoclema* contains a single species, *A*. *glaucifolium* (Franch.) W. T. Wang, endemic to the Hengduan Mountains in southwestern China [27]. Because of specific pinnatisect and penninerved leaves and spinulose pollen grains, Wang [28] proposed that *Anemone* sect. *Anemoclema* Franch. should be separated from *Anemone* as an independent genus. This treatment is widely adopted by Chinese researchers in *Floras* [21, 29, 30], checklists [31], and publications [27, 32, 33]. In contrast, non-Chinese taxonomists prefer treating this species as a monotypic section or subgenus in *Anemone* [1–3, 34–36]. However, Wang’s treatment is supported by results of karyotype and molecular phylogenies [32, 37, 38]. Furthermore, it has been documented that *Anemoclema* is close to *Clematis*, not to *Anemone* [4, 38, 39]. Therefore, *Anemoclema* has been transferred to subtribe Clematidinae [38], then subtribe Anemoninae includes *Anemone* s.l. and *Metanemone*.

To date, phylogenetic analyses of *Anemone* s.l. are mainly based on nuclear ribosomal internal transcribed spacers (nrITS) and plastid *atpB-rcbL* intergenic spacer, because the two regions show high rates of variable and parsimony-informative sites, and they are powerful to resolve phylogenies at the infrageneric level [24–26, 40, 41]. Monophyly of *Anemone* s.l. was strongly supported in these studies. However, the monophyly of *Anemone* s.l. was not resolved in other studies using other regions, but these were with limited samples [4, 39, 42]. In addition, phylogenetic relationship between subtribes Anemoninae and Clematidinae is inferred just using nrITS and *atpB-rcbL* datasets [24, 38, 41]. In this study, we extensively sampled *Hepatica* and *Pulsatilla* in subtribe Anemoninae, as well as *Anemoclema* and *Naravelia* in subtribe Clematidinae, and we sequenced nrITS, *atpB-rbcL*, and five additional plastid regions (*matK*, *rbcL*, *psbA-trnQ*, *rpoB-trnC* and *rps16*). For the *atpB-rbcL* region, we only used the intergenic spacer, so there is no overlapping with the *rbcL* gene. Based on comprehensive phylogenetic analyses, we sought to: (1) infer the phylogenetic relationships among genera within the two subtribes; (2) reevaluate the monophyly of *Anemone* s.l.; and (3) resolve the phylogenetic placement of *Anemoclema* and *Naravelia*.

## Materials and methods

### Plant samplings and ethics statement

We sampled nine of ten recognized genera in tribe Anemoneae (excluding *Kingdonia*). *Metanemone* was not sampled, because the single species *M*. *ranunculoides* has type material alone, and we were failed to collect in the field. In total, we sampled 122 accessions representing 77 species and five infraspecific taxa of tribe Anemoneae, including *Anemoclema* (1 species/6 individuals, 100% of total species, hereafter), *Anemone* (14/19, ~10%), *Archiclematis* (1/1, 100%), *Barneoudia* (3/3, 100%), *Clematis* (21/22, ~7%), *Hepatica* (9/21, ~90%), *Knowltonia* (5/5, 62.5%), *Naravelia* (6/10, 85.7%), *Oreithales* (1/2, 100%), and *Pulsatilla* (17/53, ~40%). Eleven species from five genera of Ranunculaceae (*Adonis*, *Batrachium*, *Caltha*, *Halerpestes*, and *Ranunculus*) were selected as outgroups. Silica-dried samples were collected from public land instead of protected areas in Southwestern and Western China; therefore, field permits were not required. Voucher specimens, geographic coordinates, and GenBank accessions are presented in S2 Table.

### DNA extraction, PCR and sequencing

Total genomic DNA was extracted from silica-dried leaves using modified CTAB buffer protocol. One nuclear (nrITS) and six plastid markers (*atpB-rbcL*, *matK*, *rbcL*, *psbA-trnQ*, *rpoB-trnC* and *rps16*) were amplified and sequenced. Primer information is given in S3 Table. Polymerase chain reaction (PCR) amplification for nrITS, *matK*, *psbA-trnQ*, *rbcL* and *rps*16 markers used the following protocol: one cycle 97°C for 3 min; then 33 cycles of 94°C for 50 s, 55°Cfor 50 s and 72°Cfor 60 s; and followed by 72°C for 5 min. In addition, the regions *atpB*-*rbcL* and *rpoB-trnC* were amplified using a different protocol: one cycle 80°C for 5 min; then 35 cycles of 95°C for 60 s, 50°Cfor 45 s and 65°C for 2 min; followed by 65°C for 3 min. PCR products were purified using ExoSAP-IT (Affymetrix, Santa Clara, CA, USA). Sequencing reactions were performed using the ABI Prism BigDye Terminator Kits (Applied Biosystems, Inc.) and followed the manufacturer’s protocol. Automated sequencing was performed on an ABI 3730xl DNA sequencer (Applied Biosystems).

### Phylogenetic analyses

New sequences were assembled, aligned, and adjusted using Geneious 7.0 [43]. Aligned matrices of the seven DNA regions were firstly analyzed separately, then plastid matrices were concatenated using SequenceMatrix 1.7 [44]. The DNA matrix of seven DNA regions was deposit at Figshare (DOI: 10.6084/m9.figshare.4774753). No nucleotide positions were excluded from analyses. According to the topologies of single marker datasets, monophyly of *Anemone* s.l. was recovered in nrITS and *atpB-rbcL* datasets. Previous studies using the nrITS + *atpB-rbcL* dataset well resolved the monophyly of *Anemone* s.l., therefore, the two datasets were combined in this study. To combine the plastid datasets, we did two treatments: one has all six plastid regions (i.e. six-plastid-gene dataset), and the second has five plastid regions without *atpB-rbcL* (i.e. five-plastid-gene dataset). Topological incongruence among nrITS, *atpB-rbcL*, nrITS + *atpB-rbcL* and five plastid datasets was investigated using the approximately unbiased (AU) test [45] and the Shimodaira–Hasegawa (SH) test [46]. Topologies were constrained using Mesquite 3.2 [47]. The SH and AU tests were performed using PAUP 4.0 [48].

Maximum likelihood (ML) analyses were conducted using RAxML [49]. These analyses used the GTR substitution model with gamma-distributed rate heterogeneity among sites and the proportion of invariable sites estimated from the dataset. The multiple-gene datasets were partitioned by genes. Support values for the node and clade were estimated from 1000 bootstrap replicates. ML bootstrap support (BS) values ≥ 70% were considered well supported, and BS < 50 were seen as an indication of nonsupport. Bayesian inference (BI) analyses was performed using MrBayes 3.2.6 [50], with DNA substitution models selected for each gene partition by the Bayesian information criterion (BIC) using jModeltest 2.0 [51]. Markov Chain Monte Carlo (MCMC) analyses were run in MrBayes for 10,000,000 generations for each dataset. The BI analyses were started with a random tree and sampled one tree every 1000 generations. The first 20% of the trees were discarded as burn-in, and the remaining trees were used to generate a majority-rule consensus tree. Internodes with posterior probability values (PP) ≥ 0.95 were considered as statistically significant. The best-fit model of nucleotide substitution for the seven DNA regions is listed in Table 1.

**Table 1:** Summary information of seven DNA markers. Including sequence characteristics and best-fit model of Bayesian information criterion (BIC) for Bayesian inference.

	Nuclear maker	Plastid marker						Combined dataset	
	nrITS	*atpB-rbcL*	*matK*	*psbA-trnQ*	*rbcL*	*rpoB-trnC*	*rps16*	nrITS+*atpB-rbcL*	Plastid genes (no *atpB-rbcL*)
No. of accessions/tribe Anemoneae	118/107	112/101	89/80	85/80	84/73	89/78	65/54	129/118	107/96
Aligned length (bp)	854	1266	807	806	680	1538	1055	2120	4886
Variable sites/ informative sites									
All samples	404/325	460/285	338/188	310/195	93/68	599/409	346/230	864/610	1686/1090
tribe Anemoneae	321/256	352/200	146/89	251/151	49/31	310/174	181/102	456/673	937/545
-lnL	8430.3365	6435.4960	4043.4941	4107.4513	2000.6280	7281.6526	4444.5674	—	—
*K*	239	228	182	176	170	182	134	—	—
BIC model	TIM2ef+I+G	TPM3uf+G	TPM1uf+G	TPM1uf+G	TPM1+I+G	TPM1uf+G	TPM1uf+G	—	—

## Results

### Characteristics of DNA sequences

Sequence characteristics of the DNA regions and the concatenated datasets are summarized in Table 1. For the matrix of tribe Anemoneae, the proportions of both variable site and parsimony-informative site were highest for nrITS (variable: 37.59%, and parsimony-informative: 29.98%, hereafter), followed by *psbA-trnQ* (31.14% and 18.73%), *atpB-rbcL* (27.80% and 15.80%), *rpoB-trnC* (20.16% and 11.31%), *matK* (18.09% and 11.03%), *rps16* (17.16% and 9.67%), and *rbcL* (7.21% and 4.56%). The best-fit BIC models for seven DNA regions were independent (Table 1), thus the BI analyses of the concatenated datasets were partitioned using a specific model for each DNA region.

### Phylogenetic analyses of single DNA marker

Phylogenetic relationships among genera resulting from of the seven DNA markers analyzed separately using ML and BI methods are presented in S1 Fig. As for *Barneoudia*, *Knowltonia*, and *Oreithales* only nrITS and *atpB-rbcL* sequences were available from GenBank, the three genera were not included in phylogenetic analyses of the other five plastid datasets. In addition, all samples of *Hepatica* failed to amplify for the *rps16* region.

Topologies of the seven datasets were divided into two types. The first type included nrITS and *atpB-rbcL* datasets, which supported the splitting of tribe Anemoneae into two clades, i.e. *Clematis* s.l. (including *Archiclematis* and *Naravelia*) + *Anemoclema* and *Anemone* s.l. (including *Barneoudia*, *Hepatica*, *Knowltonia*, *Oreithales*, and *Pulsatilla*). The clade *Clematis* s.l. + *Anemoclema* corresponds to a newly defined subtribe Clematidinae by Zhang et al. [38], and the clade *Anemone* s.l. corresponds to subtribe Anemoninae. The other type of dataset was the other five plastid regions. All five trees showed that *Anemoclema* was sister to *Clematis* s.l., while *Anemone* s.l. was paraphyletic. Overall, species of *Anemone* were divided into two clades in all seven trees, with one clade (*Anemone* I) close to *Pulsatilla* (not with *atpB-rbcL*), and another clade (*Anemone* II) close to *Hepatica* (but not with the nrITS and *rps16* datasets). There is no species sharing between the two *Anemone* clades. In the clade *Clematis* s.l., six datasets of single marker, except *matK* dataset, strongly supported the monophyly of *Naravelia*.

### Phylogenetic analyses of nrITS +*atpB-rbcL* dataset

**Fig 1:**
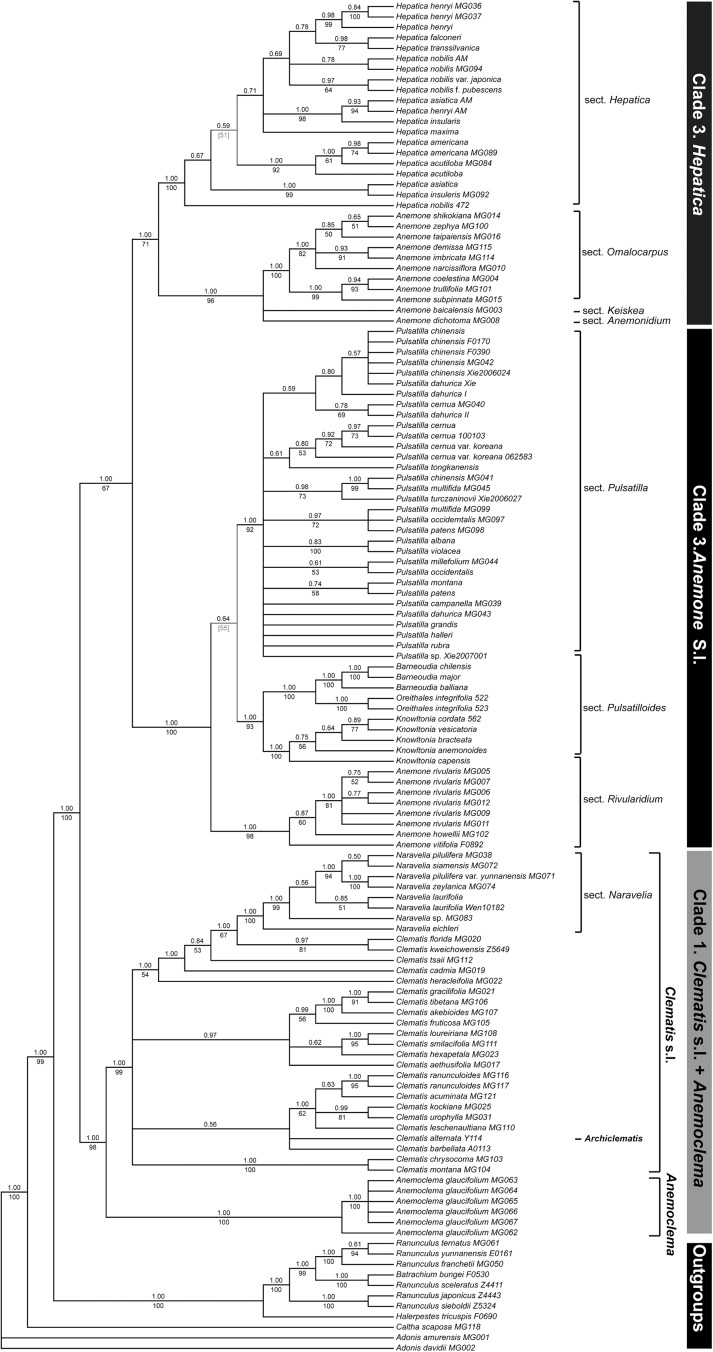
Phylogenetic relationships within tribe Anemoneae based on the combination of nrITS and *atpB-rbcL* datasets. The topology is that of the majority rule consensus of BI tree. Bootstrap values of ML are presented under branches, and posterior probability of BI above branches. Topological incongruence between ML and BI trees is indicated by colored nodes/branches, and topology of BI tree shows by dash lines with posterior probability in square bracket under branches.

**Fig 2:**
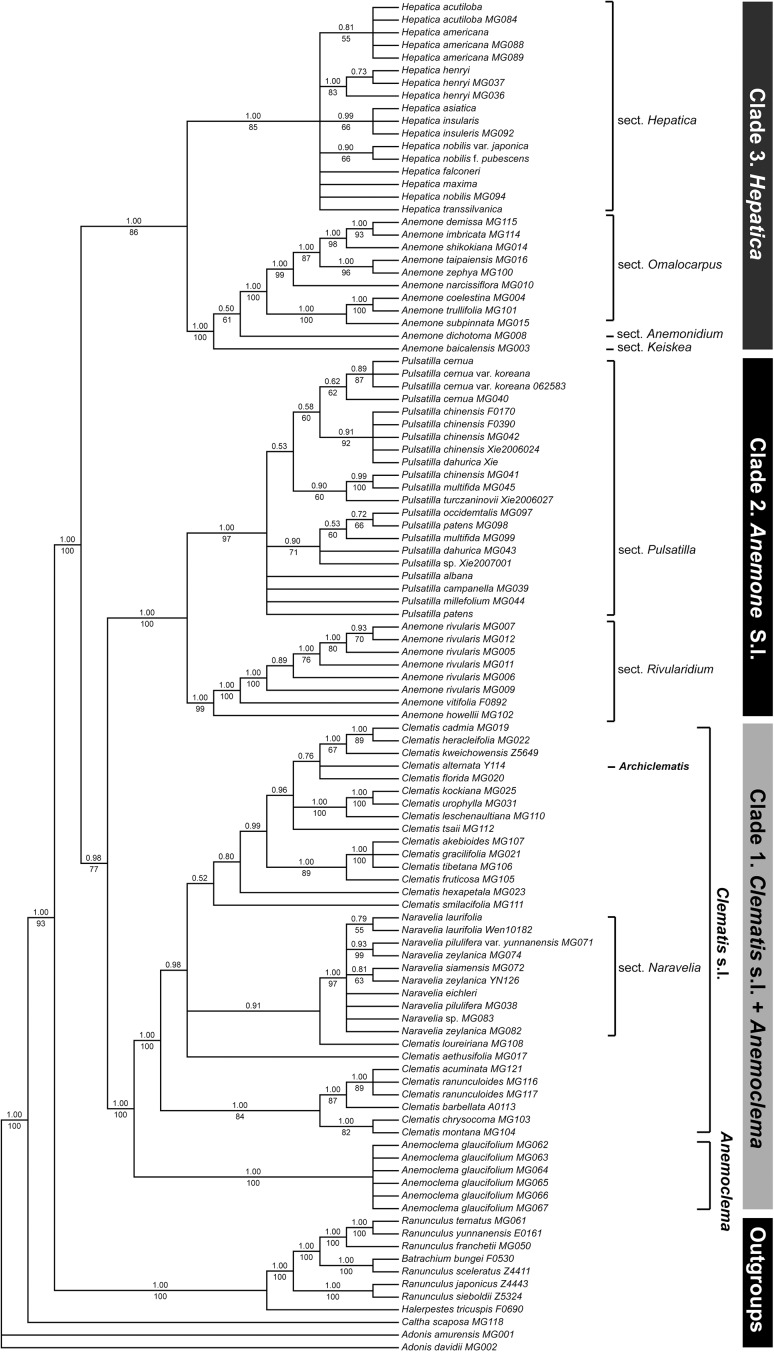
Phylogenetic relationships within tribe Anemoneae based on the combination of five-plastid-gene dataset. The five plastid genes are *matK*, *psbA-trnQ*, *rbcL*, *rpoB-trnC*, and *rps16*. The topology is that of the majority rule consensus of ML tree. Bootstrap values of ML are presented above branches, and posterior probability of BI under branches. Topological incongruence between ML and BI trees is indicated by colored nodes/branches, and topology of BI tree shows by dashed lines with posterior probability in square bracket under branches.

Topology of the combined nrITS and *atpB-rbcL* dataset is showed in Fig 1. Topological incongruence between ML and BI trees was found in two weakly resolved clades (Fig 1, S2 Fig). In the combined dataset analyses, *Clematis* s.l. + *Anemoclema* (subtribe Clematidinae, BS/PP = 98/1.00) and *Anemone* s.l. (subtribe Anemoninae, BS/PP = 67/1.00) were well supported as monophyletic. Three major clades were recognized (Fig 2): clade 1 corresponding to *Clematis* s.l. + *Anemoclema*; and clades 2 and 3 corresponding to two subgenera in *Anemone* s.l. [26]: subgenus *Anemone* and subgenus *Anemonidium*, respectively. Because subtribe Anemoninae was not supported as monophyletic by the plastid dataset (see below), we divided this subtribe into two clades to maintain consistent statements between two combined datasets.

In clade 1, both *Clematis* s.l. (BS/PP = 99/1.00) and *Anemoclema* (BS/PP = 100/1.00) are strongly supported as monophyletic. In *Anemoclema*, the Sichuan sample (MG062) was strongly supported as sister to the remaining Yunnan samples. The clade *Clematis* s.l. included *Archiclematis* and *Naravelia*. The backbone of the clade *Clematis* s.l. was poorly resolved. Four major groups were strongly supported by the BI analysis (PP > 0.95). The phylogenetic position of *Archiclematis alternata* (≡ *C*. *alternata*) was uncertain, as well as the position of *C*. *barbellata* Edgew. The monophyly of *Naravelia* (BS/PP = 100/1.00) was strongly supported, and the genus was sister to *C*. *florida* Thunb. + *C*. *kweichowensis* C. P'ei (BS/PP = 67/1.00). In the clade *Naravelia*, *N*. *eichleri* Tamura was sister to the remaining taxa, followed by an unknown species from Laos; *N*. *pilulifera* var. *yunnanensis* Y. Fei was close *N*. *zeylanica* (BS/PP = 100/1.00), but *N*. *pilulifera* Hance var. *pilulifera* was nested with *N*. *siamensis* Craib (PP = 0.50).

In clade subtribe Anemoninae (clades 2 + 3), four traditional genera (i.e., *Barneoudia*, *Hepatica*, *Knowltonia*, and *Pulsatilla*) were strongly supported as monophyletic, and *Anemone* spp. fell into two clades: *Anomene* II was close to *Hepatica* (BS/PP = 71/1.00); and *Anomene* I (sect. *Rivularidium*) was close to *Pulsatilla* in the ML analyses (BS = 51, S2 Fig), while it was close to the clade *Pulsatilla* + *Knowltonia*–*Barneoudia* (sect. *Pulsatilloides*) in the BI analysis (PP = 0.59, S2 Fig). In clades *Hepatica* and *Pulsatilla*, morphology-based species were not resolved as monophyletic yet. *Anemone* section *Omalocarpus* DC. was recovered as monophyletic in the clade *Anemone* II.

Additional ML analyses excluding samples of *Barneoudia*, *Knowltonia*, and *Oreithales* recovered three major clades (S3 Fig). In comparison with the full dataset, there is little difference in support values of the resolved clades. For example, BS value for monophyly of *Anemone* s.l. was 59 (vs. 67), that of *Anemone* II in clade 3 was 95 (vs. 96), and that of *Clematis* s.l. in clade 1 was 100 (vs. 99).

### Phylogenetic analyses of the five-plastid-gene dataset (without *atpB-rbcL*)

Phylogenetic trees of the five-plastid-gene dataset are shown in Fig 2. Topologies were consistent in both BI and ML analyses (S4 Fig). Three strongly supported clades were recognized in tribe Anemoneae, and clades were numbered following the nrITS + *atpB-rbcL* dataset. The topology resulting from this dataset was different from that of the nrITS + *atpB-rbcL* dataset in that clade 2 was nested with clade 1, *Clematis* s.l. + *Anemoclema* (BS/PP = 77/0.98). The monophyly of subtribe Anemoninae was rejected by the plastid dataset.

Three traditional genera (*Hepatica*, *Naravelia* and *Pulsatilla*) were strongly supported as monophyletic, and all six samples of *Anemoclema* formed one clade. *Clematis*, including *Naravelia*, was paraphyletic; and *Anemone* was polyphyletic, separated into two subclades, *Anemone* I in clade 2 and *Anemone* II in clade 3. Clade 3 was sister to clades 1 + 3 (BS/PP = 77/0.98). Clade 3 included two subclades, *Hepatica* (BS/PP = 93/1.00) and *Anemone* II (BS/PP = 100/1.00). Within *Hepatica*, *H*. *henryi* (BS/PP = 83/1.00) and *H*. *nobilis* (BS/PP = 66/0.90) were monophyletic, respectively. In the clade *Anemone* II, *A*. section *Omalocarpus* was recovered as monophyletic. Subsequently, clade 2 divided into two subclades, *Anemone* I and *Pulsatilla*, and phylogenetic resolution in the clade *Pulsatilla* was poor, and some of the species appeared to non-monophyletic. In clade 1, *Anemoclema* was sister to *Clematis* s.l. The clade *C*. *montana* Buch.-Ham. ex DC.–*C*. *acuminata* DC. (BS/PP = 84/1.00) was sister to the remaining *Clematis* (including *Archiclematis*) and *Naravelia*. *Clematis loureiroana* DC. was resolved as sister to *Naravelia* (PP = 0.91). Interspecific relationship in *Naravelia* was not resolved. *Clematis smilacifolia* Wall. and *C*. *hexapetala* Pall. was sister to the remaining *Clematis* (BI = 0.96), then they formed three well or strongly supported clades, *C*. *fruticosa* Turcz.*–akebioides* (Maxim.) H.J. Veitch (BS/PP = 89/1.00), *C*. *leschenaultiana*–*C*. *kockiana* C.K. Schneid. (BS/PP = 100/1.00), and *C*. *kweichowensis*–*C*. *cadmia* Buch.-Ham. ex Hook. f. & Thomson (BS/PP = 67/1.00).

### Phylogenetic analyses of the six-plastid-gene dataset

**Fig 3:**
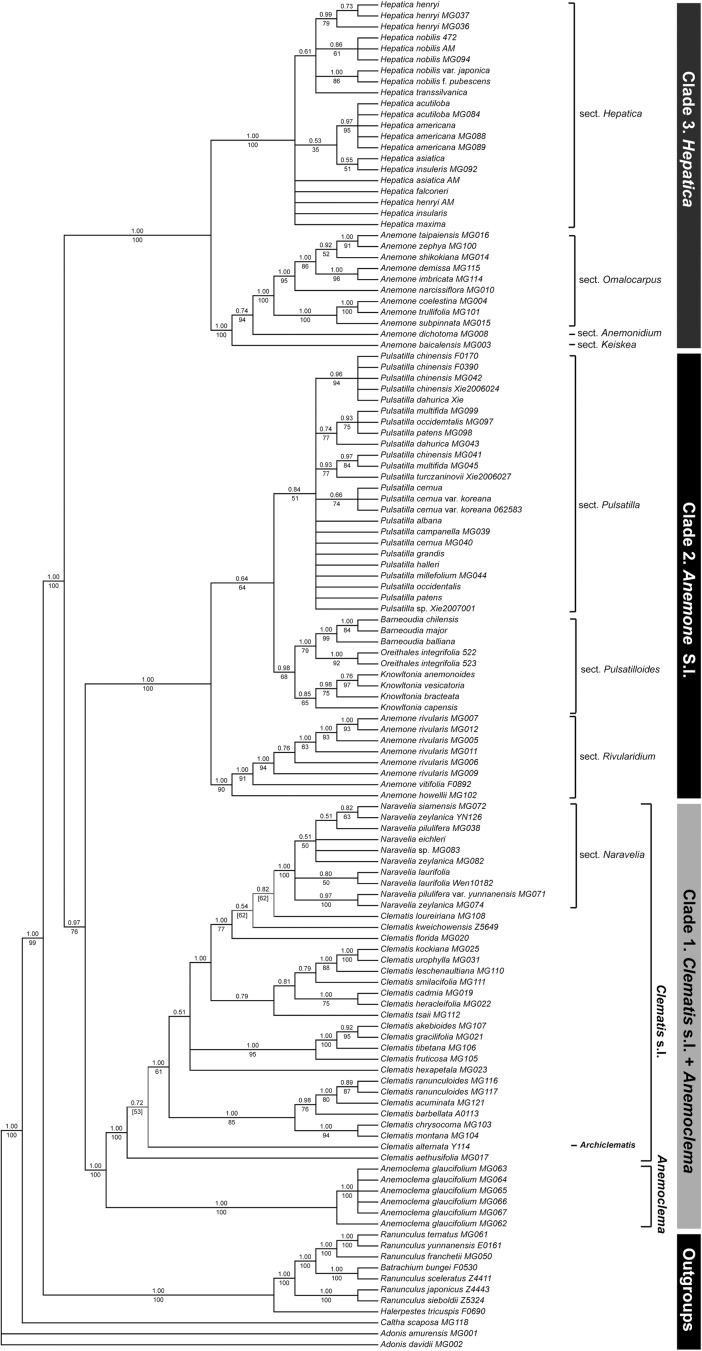
Phylogenetic relationships within tribe Anemoneae the combination of six-plastid-gene dataset. The six plastid genes are *atpB-rbcL*, *matK*, *psbA-trnQ*, *rbcL*, *rpoB-trnC*, and *rps16*. The topology is that of the majority rule consensus of ML tree. Bootstrap values of ML are presented above branches, and posterior probability of BI under branches. Topological incongruence between ML and BI trees is indicated by colored nodes/branches, and topology of BI tree shows by dash lines with posterior probability in square bracket under branches.

Topology of the six-plastid-gene dataset (Fig 3) recovered the same relationship of three major clades using five-plastid-gene dataset. However, two weakly incongruent clades between BI and ML trees were found in the clade *Clematis* s.l.: ML tree supported the clade *C*. *alternata* + *C*. *aethusifolia* Turcz. (BS = 53) and the clade *C*. *florida* + *C*. *kweichowensis* + *C*. *loureiriana* DC. (BS = 62), however, both were rejected in the BI tree (S5 Fig).

Clade 1 and clade 2 were well supported as sister (BS/PP = 76/0.97). In clade 1, *Anenoclema* was sister to *Clematis* s.l. Then, *C*. *alternata* and *C*. *aethusifolia* were sister to remaining *Clematis* spp. (BS/PP = 61/1.00), followed the clade *C*. *montana*–*C*. *ranunculoides* Franch. (BS/PP = 85/1.00). The clades *C*. *fruticosa–akebioides* (BS/PP = 89/1.00) and *C*. *leschenaultiana*–*C*. *kockiana* (BS/PP = 100/1.00) were recovered as monophyletic. *Clematis florida*, *C*. *kweichowensis* and *C*. *loureiriana* were sister to *Naravelia* (BS/PP = 77/1.00). In clade 2, three clades were the same to those in nrITS + *atpB-rbcL* dataset. The clade *Pulsatilla* was weakly supported (BS/PP = 51/0.84). The clade 3 was strongly supported by both analyses (BS/PP = 100/1.00), as well as two subclades (BS/PP = 100/1.00). In clade *Anemone* II, sect. *Omalocarpus* was recovered as monophyletic. In clade *Hepatica*, three of four samples from *H*. *henryi* formed a clade (BS/PP = 79/0.99), five samples of *H*. *nobilis* split as two groups, and two samples of *H*. *acutiloba* and three samples of *H*. *america* were sisters (BS/PP = 95/0.97).

Additional ML analyses excluding samples of *Barneoudia*, *Knowltonia*, and *Oreithales* recovered three major clades (S3 Fig). In comparison with the full dataset, there is little difference in support values of the resolved clades. For example, BS value for clades 1 + 2 was 78 (vs. 76), that of the clade *Naravelia* in clade 1 was 99 (vs. 100). One exception was that monophyly of *Pulsatilla* was strongly supported (BS = 95 vs. BS/PP = 51/0.84).

### Topological comparisons and dataset combinations

The SH and UA tests for constrained relationships using nrITS, *atpB-rbcL*, nrITS + *atpB-rbcL* and five-plastid-gene datasets are presented in Table 2. We only found that the unconstrained topology of the five-plastid dataset showed significant difference in both SH and AU tests when compared with the constraint nrITS topology, and in AU test when compared with the constrained *atpB-rbcL* topology. For combined analyses, the *atpB-rbcL* dataset was more suitable for concatenating with nrITS than the five-plastid-gene dataset, and nrITS dataset and the five-plastid-gene dataset should be analyzed separately.

**Table 2:** Summary of the Shimodaira-Hasegawa (SH) and the approximately unbiased (AU) tests. P values were less than 0.05 in boldface. Log likelihood scores for the unconstrained analysis are given, as well as the difference in log likelihood scores between the unconstrained and the constraint topologies (∂).

	Ln likelihood	∂	SH	AU
**nrITS analyses compared with constraint clades from *atpB-rbcL* and five-plastid genes analyses**				
Unconstrained nrITS analysis	9064.41715			
*atpB-rbcL*: ((A,B),((C,D),(E,(F,G))))*	9072.49361	8.07647	0.2888	0.2248
Plastid: ((C,D),((A,B),(F,(E,G))))	9074.76080	10.34366	0.2696	0.0785
***atpB-rbcL* analyses compared with constraint clades from nrITS and five-plastid gene analyses**				
Unconstrained *atpB-rbcL* analysis	6927.72870			
nrITS: ((A,B),(C,(D,(F,(E,G))))))	6934.32995	6.60125	0.38310	0.2220
Plastid I: ((C,D),((A,B),(E,(F,G))))	6931.46488	3.73618	0.53430	0.2301
Plastid II: ((C,D),((A,B),(F,(E,G))))	6929.99927	2.27058	0.64490	0.5139
**nrITS + *atpB-rbcL* analyses compared with constraint clades from five-plastid gene analyses**				
Unconstrained nrITS + *atpB-rbcL* analysis	16844.75792			
Plastid I: ((C,D),((A,B),(E,(F,G))))	16862.85264	18.09472	0.2150	0.1165
Plastid II: ((C,D),((A,B),(F,(E,G))))	16861.05424	16.29632	0.1137	0.0588
**Five-plastid-gene analyses compared with constraint clades from nrITS and *atpB-rbcL* analyses**				
Unconstrained five-plastid-gene analysis	23900.91280			
nrITS: ((A,B),(C,(D,(E,G))))	23911.26222	80.32224	**0.0001**	**0.0000**
*atpB-rbcL*: ((A,B),((C,D),(E,G)))	23981.23504	10.34943	0.3485	**0.0296**

*Notes: A, *Anemoclema*; B. *Clematis* s.l.; C. *Hepatica*; D, *Anemone* II; E. *Anemone* I; F, (*Knowltonia*, (*Barneoudia*, *Oreithales*)); G, *Pulsatilla*.

## Discussion

### Phylogenetic incongruence among datasets

Monophyly of tribe Anemoneae was strongly supported by seven single marker datasets (S1 Fig). Within tribe Anemoneae, five major groups were recognized in all seven datasets, six major groups in the six datasets (except *rps16* dataset), and nine major groups in both nrITS and *atpB-rbcL* datasets. Species of *Barneoudia*, *Knowltonia* and *Oreithales* were absent from the *psbA-trnQ*, *rbcL rpoB-trnC* and *rps16* datasets, and *Hepatica* from the *rps16* dataset because we failed to generate sequences from the samples, or there was no sequence in GenBank. For the five datasets, the remaining major groups were well supported as monophyletic. Overall, phylogenetic resolution of the backbone was poor using the single marker datasets (S1 Fig), and relationships among groups were incongruent. Based on the similarity of topologies, and the SH and AU tests, the seven datasets tended to split in two groups: one group included nrITS and *atpB-rbcL*, and the other group included the remaining five plastid datasets. We confirmed that taxa sampling had no effect on backbone relationships obtained with either the nrITS or *atpB-rbcL* datasets, because clades *Clematis* + *Anemoclema* and *Anemone* s.l. were also supported when *Barneoudia*, *Knowltonia* and *Oreithales* were excluded (S3 Fig). Generally, the conflicting topologies in plants are found between nuclear and plastid datasets [52–56]. In tribe Anemoneae, the topologies based on the nrITS and *atpB-rbcL* datasets were consistent [26, 41, 57]. However, topological incongruence was found between the five-plastid-dataset and *atpB-rbcL* suggested that plastid genes may be evolved independently in tribe Anenomeae. In a large-scale analysis, Zeng et al. [58] have documented that topologies showed differences between the single copy region genes and inverted repeat region genes, because genes in the inverted repeated region are more conservative than those in the single copy region. Meanwhile, the coding genes are more conservative than the non-coding genes. In this study, six plastid genes were not powerful enough to clarify this question. Based on published plastomes of Ranunculaceae, at least two large rearrangements (*rps4* CDS and *trnH* tRNA- *rps16* CDS) were found tribe Anenomeae, which has been detected using restriction enzymes [59]. As more and more chloroplast genomes are published [60], comparative analyses of whole chloroplast genomes may help to understand the evolutionary history of plastid genes.

Compared to the single marker datasets, phylogenetic resolution was significantly improved when the nrITS dataset was combined with the *atpB-rbcL* dataset, and five plastid datasets were concatenated. Meanwhile, phylogenetic conflicts between the two combined datasets became significant (AU test: *P* = 0.0588). In the topology, monophyly of subtribe Anemoninae was well supported by the nrITS + *atpB-rbcL* dataset; whereas subtribe Anemoninae was paraphyletic using the plastid dataset. In addition, support values for the clades 1 + 2 were not increased yet when the *atpB-rbcL* dataset was combined with the other five plastid datasets. The AU test indicated that the *atpB*-*rbcL* and the five-plastid gene datasets were tended to analyze separately.

### Phylogenetic placement of *Anemoclema* and *Naravelia*

*Anemoclema* is upgraded as an independent genus primarily based morphological characters [28]. The flowers of *Anemoclema glaucifolium* resemble to *Anemone*, and its persistent styles with hairs to *Pulsatilla* [28]. Therefore, *Anemoclema* should belong to *Anemone* s.l or subtribe Anemoninae. However, preliminary phylogenetic analyses show that *Anemoclema* is the sister to *Clematis* + *Naravelia*, while *Anemone* and *Pulsatilla* form another clade [4]. Due to the study of Wang et al. [4] focusing on resolving the relationships of Ranunculales, *Anemoclema* and the other three genera (*Anemone*, *Clematis* and *Pulsatilla*) only included one sample/species. Subsequently, Zhang et al. [38] sampled multiple species of *Anemone*, *Clematis*, and *Pulsatilla*, and three individuals of *Anemoclema*, and they sequenced the nrITS and *atpB-rbcL* regions. Their results strongly support the transfer of *Anemoclema* to subtribe Clematidinae. In this study, we sampled six individuals of *Anemoclema* representing its whole distribution regions in southwestern China, and 18 taxa of *Pulsatilla*, and sequenced nrITS and six plastid regions. Phylogenetic analyses revealed that seven single marker datasets and three combined datasets all recovered the clade *Anemoclema* + *Clematis* s.l. Therefore, *Anemoclema* is clearly excluded from *Anemone* s.l. or subtribe Anemoninae as a distinctive genus that is sister to *Clematis* s.l.

Morphological delimitation of the genus *Clematis* is very controversial, several small genera have been proposed [12]. Of these genera, *Naravelia* is widely accepted as an independent genus [2, 3, 18, 21, 29, 61], although it is subsumed within *Clematis* s.l. by some taxonomists [14, 22, 62]. *Naravelia* is separated from *Clematis* as an independent genus by having narrow and long petals and leaflet tendrils. Traditionally, *Clematis* section *Atragene* (L.) DC. is supposed to have petals. However, floral development has shown that petals in *Clematis macropetala* are initiated from stamen primordia, and then antherless filaments expand to petal-like staminodia [63]. Therefore, we suggested that the “petals” of *Naravelia* may be the narrow and long staminodia.

Miikeda at al. [19] firstly revealed that *Naravelia* was nested with *Clematis*, then *N*. *laurifolia* and *N*. *eichleri* formed a clade. Subsequent studies [20, 24, 37, 39] confirmed the result of Miikeda at al. [19] because they used same/similar dataset of *Naravelia* from GenBank, or sequenced the same species. Based on our extensive sampling of *Naravelia*, we recovered the monophyly of *Naravelia* (including *N*. *eichleri*), which should be treated as a subgenus or section. *Naravelia eichleri* was originally placed in *Naravelia* by Tamura [18] based on fruiting and imperfect specimens, then Tamura [64] himself transferred it to *Clematis* after he collected fertile specimens without petals and leaflet tendrils. However, the sequenced sample of *N*. *eichleri* was collected by Tamura from Thailand [19]. In the present study, we demonstrated that *N*. *eichleri* was included the *Naravelia* group. The nrITS + *atpB-rbcL* dataset strongly supported *N*. *eichleri* as sister to remaining species of *Naravelia*, indicating that species with petal-like staminodia and leaflet tendrils may be derived from an ancient without staminodia and leaflet tendrils only once.

### Generic delimitation in subtribe Anemoninae

According to molecular phylogenies [25, 26, 41, 65], *Barneoudia*, *Hepatica*, *Knowltonia*, *Oreithales*, and *Pulsatilla* were suggested to subsumed with *Anemone*. When *Anemoclema* has transferred to subtribe Clematidinae [38], current subtribe Anemoninae includes *Anemone* s.l. and *Metanemone*. To date, the only species of *Metanemone*, *M*. *ranunculoides* W. T. Wang, was collected only one time from the type locality in Weixi County, northwestern Yunnan. There is no sample of *Metanemone* included in any phylogenetic analyses, so the systematic placement of this genus remains unclear.

*Anemone* s.l. has been suggested to include *Barneoudia*, *Hepatica*, *Knowltonia*, *Oreithales*, and *Pulsatilla*, because this group is strongly supported as monophyletic by the combined nrITS and *atpB-rbcL* dataset [25, 26, 41, 65]. Our phylogenetic analyses also recovered the monophyly of *Anemone* s.l. using nrITS + *atpB-rbcL* dataset. Based on 26S rDNA and other three plastid markers (*matK*, *rbcL*, *trnL-F*), however, Wang et al. [4] revealed that the clade *Pulsatilla* + *Anemone* was nested with *Clematis* s.l., and that *Hepatica* was the sister to them. This conflicting result might be caused by limited sampling from tribe Anemoneae [26]. Nevertheless, the concatenated plastid dataset with extensive sampling of this tribe also revealed the paraphyly of *Anenome* s.l. in this study. Therefore, *Barneoudia*, *Knowltonia*, *Oreithales*, and *Pulsatilla* in clade 2 are strongly supported to subsume with *Anemone* s.l. [26], whereas *Hepatica* and *Anemone* II in clade 3 tends to be treated as an independent genus, i.e. *Hepatica*. The clade 3 corresponds to subgenus *Anemonidium* (Spach) Juz. [23, 26], which is characterized by a chromosome number equal to 7; achenes are globose (usually wider than long) and nearly glabrous (or with short, straight hairs) with thick walls; and each head may yield no more than 50 achenes.

### Recommendations for reclassification of tribe Anemoneae

Morphologically, two subtribes have been recognized in tribe Anemoneae [1, 21]. Subtribe Anemoninae is characterized by erect herbs with basal leaves and imbricate sepals, and subtribe Clematidinae by lianas with opposite leaves (except *Archiclematis alternata*) and valvate sepals. However, *Anemoclema*, an Anemoninae-type genus, tends to transfer to subtribe Clematidinae [38]. When this treatment was adopted, diagnostic characters between subtribes Anemoninae and Clematidinae became confused. Moreover, the concatenated plastid datasets have demonstrated that subtribe Anemoninae is paraphyletic. Therefore, the subtribe rank in this tribe becomes inapplicable, and it should be abolished in future classifications.

*Clematis* s.l. is strongly supported as monophyletic in all phylogenetic analyses [19, 20, 24]. Therefore, *Archiclematis* and *Naravelia* must be subsumed with *Clematis* [20, 22]. Because phylogenetic resolution within *Clematis* s.l. is poor, morphology-based infrageneric classifications are not supported [19, 20]. Phylogenetic placements of *Archiclematis* and *Naravelia* are not resolved; however, monophyly of *Naravelia* is strongly supported. According to previous morphological classification, we suggested that *Archiclematis* and *Naravelia* should be conservatively retained as sections in *Clematis* [14, 66, 67].

Phylogenetic conflicts between nrITS + *atpB-rbcL* and the concatenated plastid datasets for *Anemone* s.l. provide new clues to redefine generic boundaries in this group. Phylogenetic clustering integrating morphological delimitations tend to split *Anemone* s.l. into two genera. Subgenus *Anemone*, defined by Hoot et al. [23, 26], corresponds to the new *Anemone* s.l., including *Barneoudia*, *Knowltonia*, *Oreithales*, and *Pulsatilla*. This genus includes four sections: *Anemone*, *Rivularisium*, *Pulsatilla*, and *Pusatilloides* [23, 26]. The subgenus *Anemoniudium* (Spach) Juz. needs to be separated as an independent genus, *Hepatica*. In the new genus *Hepetica*, four sections were recognized, *Hepatica* Spreng, *Anemonidium* Spach, *Keiska* Tamura, and *Omalocarpus* DC. [23, 26].

## Conclusions

Monophyly of tribe Anemoneae has been demonstrated by several studies [4, 8–11]. However, phylogenetic relationship among genera was not full resolved, due to limited DNA markers were used, and/or incomplete genera samplings were analyzed. In this study, we included nine of ten recognized genera in tribe Anemoneae (only *Metanemone* was not sampled) and used one nuclear and six plastid markers to reconstruct a comprehensive phylogeny of tribe Anemoneae. Based on evaluation of topological incongruence, seven DNA markers were classified as two groups, nrITS and *atpB-rbcL*, and the remaining five plastid genes. The combined datasets resolved tribe Anemoneae as three major clades: clade 1 included *Anemoclema* and *Clematis* s.l. (including *Archiclematis* and *Naravelia*), clades 2 and 3 corresponded to *Anemone* subgenus *Anemone* (including *Barneoudia*, *Knowltonia*, *Oreithales*, and *Pulsatilla*), and subgenus *Anemonidium* (including *Hepatica*), respectively. The nrITS + *atpB-rbcL* supported the monophyletic of *Anomone* s.l. (including clades 2 and 3). However, the five-plastid-gene dataset made subgenus *Anemone* (clade 2) sister to the clade *Anemoclema* + *Clematis* s.l. (clade 1). Our results strongly supported to subsume *Archiclematis* and *Naravelia* within *Clematis* s.l., and to retain *Anemoclema* as an independent genus. For the genus *Anemone* s.l., all analyses supported to include *Barneoudia*, *Knowltonia*, *Oreithales*, and *Pulsatilla* in this genus. However, the five-plastid-gene dataset tended to retain *Hepatica* as a separated genus, corresponding to *Anemone* subgenus *Anemonidium*. Therefore, the updated tribe Anemoneae consists of four revised genera, *Anemoclema*, *Anemone* s.l., *Clematis* s.l. and *Hepatica*, and an unresolved genus, *Metanemone*.

## Supporting information

S1 Table Summary of classifications in tribe Anemoneae. (XLSX)

S2 Table Voucher information and NCBI accessions of studied samples. Note: TBD, accession number of new sequences to be determined by GenBank.
(XLSX)

S3 Table Primer information for PCR and sequencing. (DOC)

S1 Fig ML and BI trees inferred from individual dataset of the seven DNA markers. Topology shows the majority rule consensus of ML tree. Topological incongruence between ML and BI trees are indicated by colored nodes/branches and posterior probability in square bracket under branches.
(PDF)

S2 Fig Phylogram of ML trees using nrITS + *atpB-rbcL* and six-plastid-gene datasets by excluding *Barneoudia*, *Knowltonia*, and *Oreithales*. (PDF)

S3 Fig Phylogram of ML and BI trees using nrITS + *atpB-rbcL* dataset. (PDF)

S4 Fig Phylogram of ML and BI trees using the five-plastid-gene dataset. (PDF)

S5 Fig Phylogram of ML and BI trees using the six-plastid-gene dataset. (PDF)
