# Supplementary material for: Phylogenetic reassessment of tribe Anemoneae (Ranunculaceae): Non-monophyly of Anemone s.l. revealed by plastid datasets
Source: PLoS One. 2017 Mar 31;12(3):e0174792. doi: 10.1371/journal.pone.0174792 (PMC5376084; doi:10.1371/journal.pone.0174792)
Supplement: S3 Table — (DOC) [file pone.0174792.s003.doc]

**Table S2.** Primer information for PCR and sequencing

| DNA marker | | Primer sequence (5′ to 3′) | | References | |  |
| --- | --- | --- | --- | --- | --- | --- |
|  | nrITS | | 18sF: ACGAATTCAAGGTCCGGTGAAGTGTTCG  26sR: TAGAATTCCCCGGTTCGCTCGCCGTTAC | | Gruenstaeu*dl et a*l. (2009) | |
|  |  | | AB101: ACGAATTCAAGGTCCGGTGAAGTGTTC  AB102: GTAGAATTCCCCGGTTCGCTCGCCGTTAC | | Miike*da et a*l. (2006) | |
|  | *atpB-rbcL* | | atpBF: AGTAGTAGGATTGATTCTCA  rbcLR: CAACACTTGCTTTAGTCTCT | | Miike*da et a*l. (2006) | |
|  | *matK* | | 1R_KIM: CGTACAGTACTTTTGTGTTTACGAG  3F_KIM: CCCAGTCCATCTGGAAATCTTGGTTC | | Hollingswor*th et a*l. (2009) | |
|  | *rbcL* | | 1F: ATGTCACCACAAACAGAAAC  724R: TCGCATGTACCTGCAGTAGC | | F*ay et a*l. (1997) | |
|  | *rpoB-trnC* | | rpoBF: CCTTGATCAATGAACCTACAAAATC  trnCR: ATTTGCAGTCCTCTGCCTTAC  CSPF: ATCACTTATCCCAGTTCCAC  CSPR: CTTATTTATATATGAGATGCCCACA | | Miike*da et a*l. (2006) | |
|  | *rps16* | | Rpsf: GTGGTAGAAAGCAACGTGCGACTT  Rpsr2: TCGGGATCGAACATCAATTGCAAC | | Oxelm*an et a*l. (1997) | |
|  | *psbA-trnQ* | | psbAF: GCTCACAACTTCCCTTTAGA  trnQR: TGGCCAAGTGGTAAGGCA | | Miike*da et a*l. (2006) | |
|  |  | | psbAF: GTTATGCATGAACGTAATGCTC  trnH2: CGCGCATGGTGGATTCACAATCC | | Sa*ng et a*l. (1997)  Tate and Simpson (2003) | |

**Literature cited**

**Fay MF, Swensen SM, Chase MW.** **1997.** Taxonomic affinities of *Medusagyne oppositifolia* (Medusagynaceae). *Kew Bulletin* **52:** 111-120.

**Gruenstaeudl M, Urtubey E, Jansen RK, et al.** **2009.** Phylogeny of Barnadesioideae (Asteraceae) inferred from DNA sequence data and morphology. *Molecular Phylogenetics and Evolution* **51:** 572-587.

**Hollingsworth ML, Clark AA, Forrest LL, et al.** **2009.** Selecting barcoding loci for plants: evaluation of seven candidate loci with species-level sampling in three divergent groups of land plants. *Molecular Ecology Resources* **9:** 439-457.

**Miikeda O, Kita K, Handa T, et al.** **2006.** Phylogenetic relationships of *Clematis* (Ranunculaceae) based on chloroplast and nuclear DNA sequences. *Botanical Journal of the Linnean Society* **152:** 153-168.

**Oxelman B, Lid, xe, et al.** **1997.** Chloroplast rps16 intron phylogeny of the tribe Sileneae (Caryophyllaceae). *Plant Systematics and Evolution* **206:** 393-410.

**Sang T, Crawford D, Stuessy T.** **1997.** Chloroplast DNA phylogeny, reticulate evolution, and biogeography of *Paeonia* (Paeoniaceae). *American Journal of Botany* **84:** 1120.

**Tate JA, Simpson BB.** **2003.** Paraphyly of *Tarasa* (Malvaceae) and diverse origins of the polyploid species. *Systematic Botany* **28:** 723-737.
