# Supplementary material for: Phylogenetic reassessment of tribe Anemoneae (Ranunculaceae): Non-monophyly of Anemone s.l. revealed by plastid datasets
Source: PLoS One. 2017 Mar 31;12(3):e0174792. doi: 10.1371/journal.pone.0174792 (PMC5376084; doi:10.1371/journal.pone.0174792)
Supplement: S1 Fig — Topology shows the majority rule consensus of ML tree. Topological incongruence between ML and BI trees are indicated by colored nodes/branches and posterior probability in square bracket under branches. (PDF) [file pone.0174792.s004.pdf]

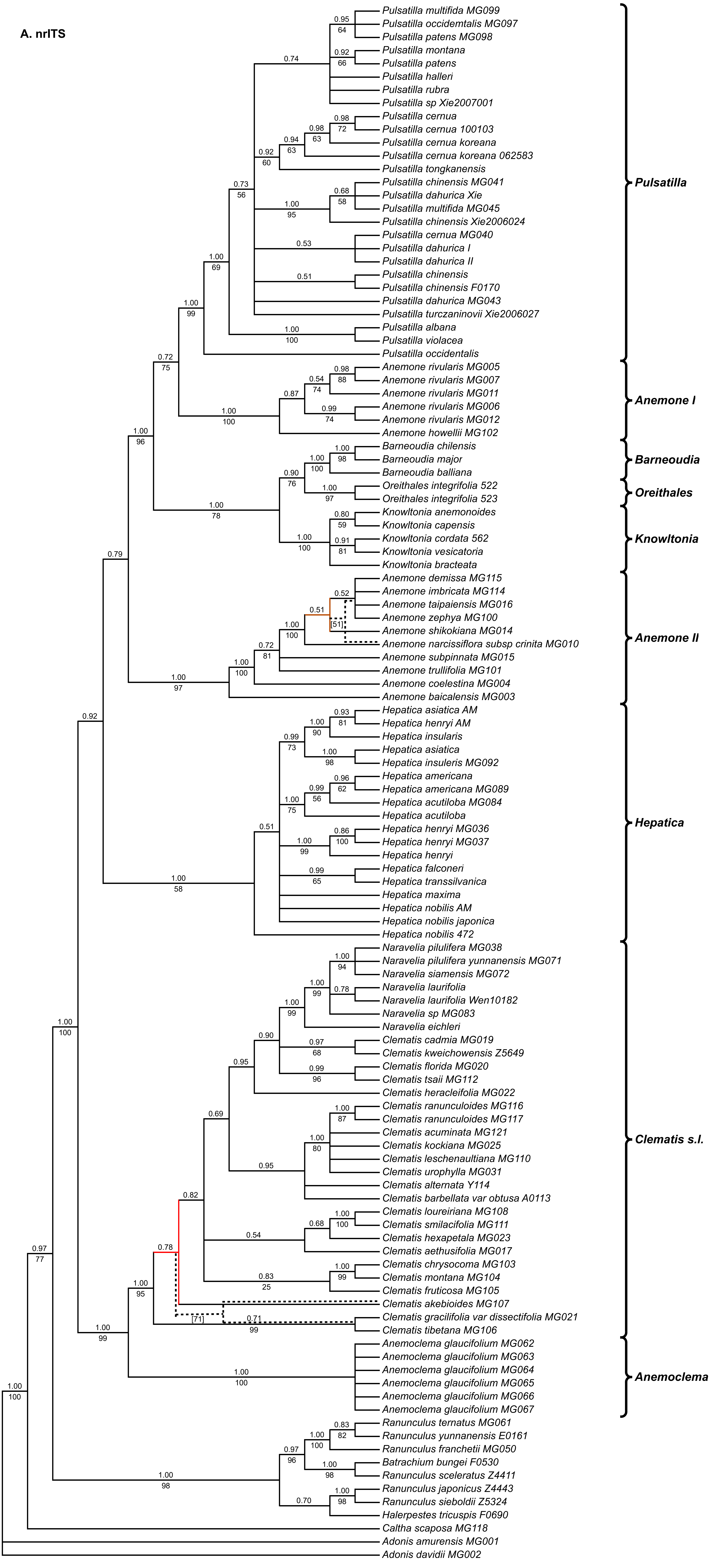
A. nrITS
Pulsatilla multifida MG099
Pulsatilla occidemtalis MG097
Pulsatilla patens MG098
Pulsatilla montana
Pulsatilla patens
Pulsatilla halleri
Pulsatilla rubra
Pulsatilla sp Xie2007001
Pulsatilla cernua
Pulsatilla cernua 100103
Pulsatilla cernua koreana
Pulsatilla cernua koreana 062583
Pulsatilla tongkanensis
Pulsatilla chinensis MG041
Pulsatilla dahurica Xie
Pulsatilla multifida MG045
Pulsatilla chinensis Xie2006024
Pulsatilla cernua MG040
Pulsatilla dahurica I
Pulsatilla dahurica II
Pulsatilla chinensis
Pulsatilla chinensis F0170
Pulsatilla dahurica MG043
Pulsatilla turczaninovii Xie2006027
Pulsatilla albana
Pulsatilla violacea
Pulsatilla occidentalis
Anemone rivularis MG005
Anemone rivularis MG007
Anemone rivularis MG011
Anemone rivularis MG006
Anemone rivularis MG012
Anemone howellii MG102
Barneoudia chilensis
Barneoudia major
Barneoudia balliana
Oreithales integrifolia 522
Oreithales integrifolia 523
Knowltonia anemonoides
Knowltonia capensis
Knowltonia cordata 562
Knowltonia vesicatoria
Knowltonia bracteata
Anemone demissa MG115
Anemone imbricata MG114
Anemone taipaiensis MG016
Anemone zephya MG100
Anemone shikokiana MG014
Anemone narcissiflora subsp crinita MG010
Anemone subpinnata MG015
Anemone trullifolia MG101
Anemone coelestina MG004
Anemone baicalensis MG003
Hepatica asiatica AM
Hepatica henryi AM
Hepatica insularis
Hepatica asiatica
Hepatica insuleris MG092
Hepatica americana
Hepatica americana MG089
Hepatica acutiloba MG084
Hepatica acutiloba
Hepatica henryi MG036
Hepatica henryi MG037
Hepatica henryi
Hepatica falconeri
Hepatica transsilvanica
Hepatica maxima
Hepatica nobilis AM
Hepatica nobilis japonica
Hepatica nobilis 472
Naravelia pilulifera MG038
Naravelia pilulifera yunnanensis MG071
Naravelia siamensis MG072
Naravelia laurifolia
Naravelia laurifolia Wen10182
Naravelia sp MG083
Naravelia eichleri
Clematis cadmia MG019
Clematis kweichowensis Z5649
Clematis florida MG020
Clematis tsaii MG112
Clematis heracleifolia MG022
Clematis ranunculoides MG116
Clematis ranunculoides MG117
Clematis acuminata MG121
Clematis kockiana MG025
Clematis leschenaultiana MG110
Clematis urophylla MG031
Clematis alternata Y114
Clematis barbellata var obtusa A0113
Clematis loureiriana MG108
Clematis smilacifolia MG111
Clematis hexapetala MG023
Clematis aethusifolia MG017
Clematis chrysocoma MG103
Clematis montana MG104
Clematis fruticosa MG105
Clematis akebioides MG107
Clematis gracilifolia var dissectifolia MG021
Clematis tibetana MG106
Anemoclema glaucifolium MG062
Anemoclema glaucifolium MG063
Anemoclema glaucifolium MG064
Anemoclema glaucifolium MG065
Anemoclema glaucifolium MG066
Anemoclema glaucifolium MG067
Ranunculus ternatus MG061
Ranunculus yunnanensis E0161
Ranunculus franchetii MG050
Batrachium bungei F0530
Ranunculus sceleratus Z4411
Ranunculus japonicus Z4443
Ranunculus sieboldii Z5324
Halerpestes tricuspis F0690
Caltha scaposa MG118
Adonis amurensis MG001
Adonis davidii MG002
Pulsatilla
Anemone I
Barneoudia
Oreithales
Knowltonia
Anemone II
Hepatica
Clematis s.l.
Anemoclema

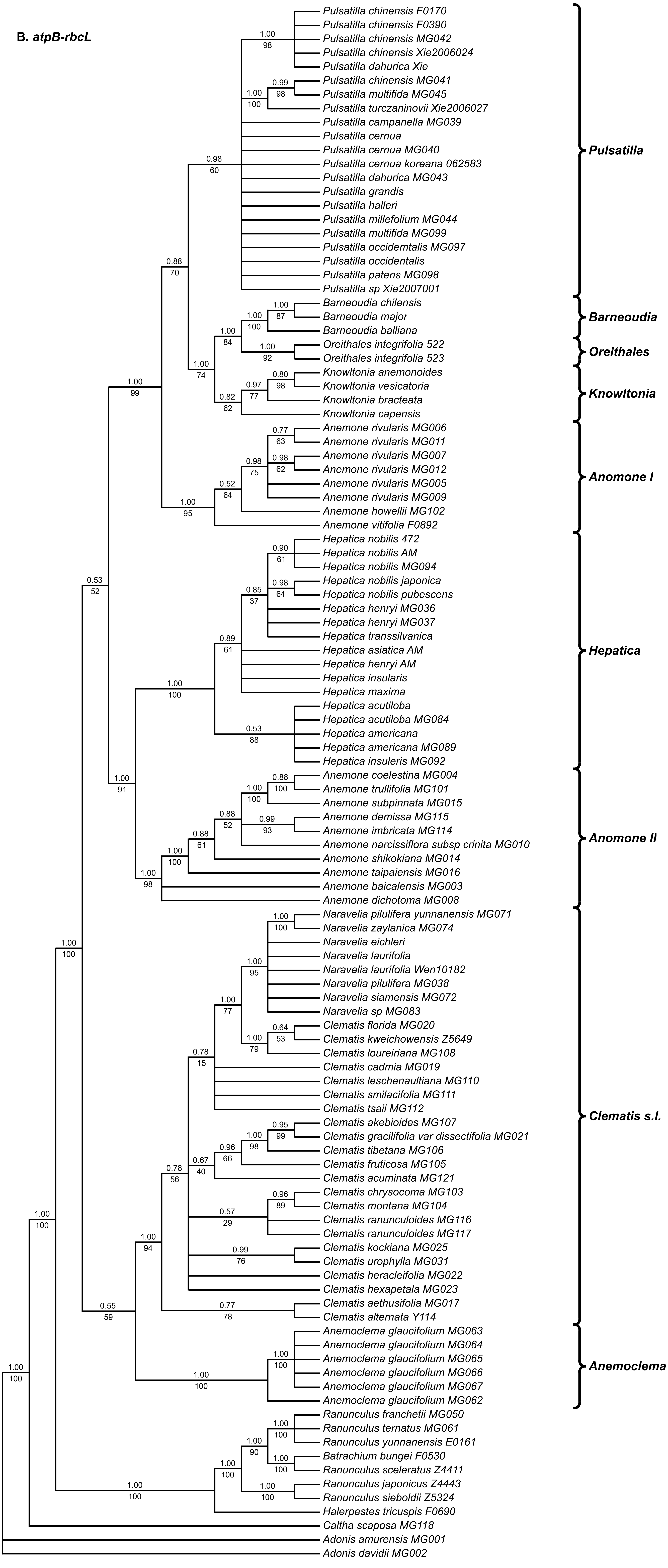
B. atpB-rbcL
Pulsatilla chinensis F0170
Pulsatilla chinensis F0390
Pulsatilla chinensis MG042
Pulsatilla chinensis Xie2006024
Pulsatilla dahurica Xie
Pulsatilla chinensis MG041
Pulsatilla multifida MG045
Pulsatilla turczaninovii Xie2006027
Pulsatilla campanella MG039
Pulsatilla cernua
Pulsatilla cernua MG040
Pulsatilla cernua koreana 062583
Pulsatilla dahurica MG043
Pulsatilla grandis
Pulsatilla halleri
Pulsatilla millefolium MG044
Pulsatilla multifida MG099
Pulsatilla occidemtalis MG097
Pulsatilla occidentalis
Pulsatilla patens MG098
Pulsatilla sp Xie2007001
Barneoudia chilensis
Barneoudia major
Barneoudia balliana
Oreithales integrifolia 522
Oreithales integrifolia 523
Knowltonia anemonoides
Knowltonia vesicatoria
Knowltonia bracteata
Knowltonia capensis
Anemone rivularis MG006
Anemone rivularis MG011
Anemone rivularis MG007
Anemone rivularis MG012
Anemone rivularis MG005
Anemone rivularis MG009
Anemone howellii MG102
Anemone vitifolia F0892
Hepatica nobilis 472
Hepatica nobilis AM
Hepatica nobilis MG094
Hepatica nobilis japonica
Hepatica nobilis pubescens
Hepatica henryi MG036
Hepatica henryi MG037
Hepatica transsilvanica
Hepatica asiatica AM
Hepatica henryi AM
Hepatica insularis
Hepatica maxima
Hepatica acutiloba
Hepatica acutiloba MG084
Hepatica americana
Hepatica americana MG089
Hepatica insuleris MG092
Anemone coelestina MG004
Anemone trullifolia MG101
Anemone subpinnata MG015
Anemone demissa MG115
Anemone imbricata MG114
Anemone narcissiflora subsp crinita MG010
Anemone shikokiana MG014
Anemone taipaiensis MG016
Anemone baicalensis MG003
Anemone dichotoma MG008
Naravelia pilulifera yunnanensis MG071
Naravelia zaylanica MG074
Naravelia eichleri
Naravelia laurifolia
Naravelia laurifolia Wen10182
Naravelia pilulifera MG038
Naravelia siamensis MG072
Naravelia sp MG083
Clematis florida MG020
Clematis kweichowensis Z5649
Clematis loureiriana MG108
Clematis cadmia MG019
Clematis leschenaultiana MG110
Clematis smilacifolia MG111
Clematis tsaii MG112
Clematis akebioides MG107
Clematis gracilifolia var dissectifolia MG021
Clematis tibetana MG106
Clematis fruticosa MG105
Clematis acuminata MG121
Clematis chrysocoma MG103
Clematis montana MG104
Clematis ranunculoides MG116
Clematis ranunculoides MG117
Clematis kockiana MG025
Clematis urophylla MG031
Clematis heracleifolia MG022
Clematis hexapetala MG023
Clematis aethusifolia MG017
Clematis alternata Y114
Anemoclema glaucifolium MG063
Anemoclema glaucifolium MG064
Anemoclema glaucifolium MG065
Anemoclema glaucifolium MG066
Anemoclema glaucifolium MG067
Anemoclema glaucifolium MG062
Ranunculus franchetii MG050
Ranunculus ternatus MG061
Ranunculus yunnanensis E0161
Batrachium bungei F0530
Ranunculus sceleratus Z4411
Ranunculus japonicus Z4443
Ranunculus sieboldii Z5324
Halerpestes tricuspis F0690
Caltha scaposa MG118
Adonis amurensis MG001
Adonis davidii MG002
Pulsatilla
Barneoudia
Oreithales
Knowltonia
Anomone I
Hepatica
Anomone II
Clematis s.l.
Anemoclema

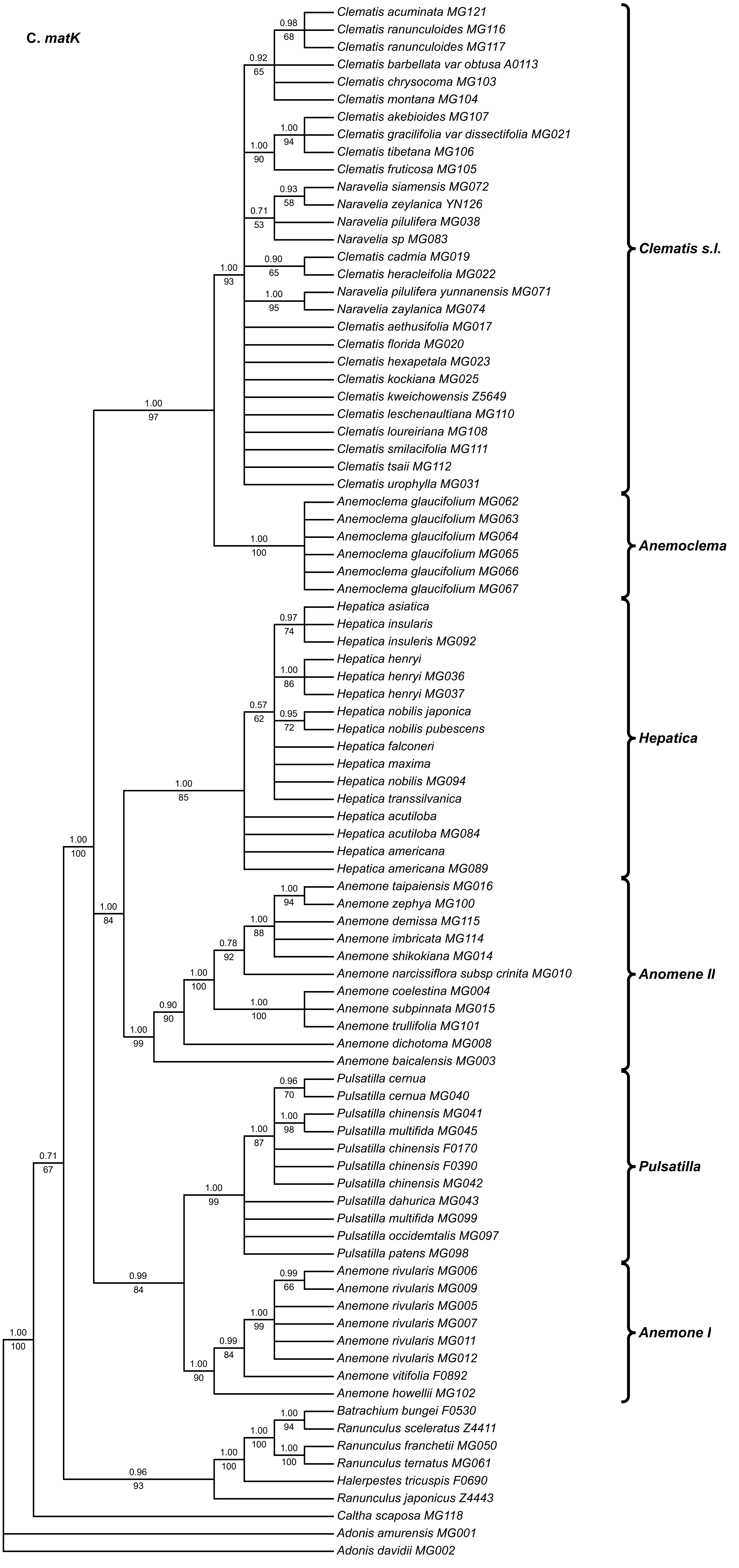

C. matK
Clematis acuminata MG121
Clematis ranunculoides MG116
Clematis ranunculoides MG117
Clematis barbellata var obtusa A0113
Clematis chrysocoma MG103
Clematis montana MG104
Clematis akebioides MG107
Clematis gracilifolia var dissectifolia MG021
Clematis tibetana MG106
Clematis fruticosa MG105
Naravelia siamensis MG072
Naravelia zeylanica YN126
Naravelia pilulifera MG038
Naravelia sp MG083
Clematis cadmia MG019
Clematis heracleifolia MG022
Naravelia pilulifera yunnanensis MG071
Naravelia zaylanica MG074
Clematis aethusifolia MG017
Clematis florida MG020
Clematis hexapetala MG023
Clematis kockiana MG025
Clematis kweichowensis Z5649
Clematis leschenaultiana MG110
Clematis loureiriana MG108
Clematis smilacifolia MG111
Clematis tsaii MG112
Clematis urophylla MG031
Anemoclema glaucifolium MG062
Anemoclema glaucifolium MG063
Anemoclema glaucifolium MG064
Anemoclema glaucifolium MG065
Anemoclema glaucifolium MG066
Anemoclema glaucifolium MG067
Hepatica asiatica
Hepatica insularis
Hepatica insuleris MG092
Hepatica henryi
Hepatica henryi MG036
Hepatica henryi MG037
Hepatica nobilis japonica
Hepatica nobilis pubescens
Hepatica falconeri
Hepatica maxima
Hepatica nobilis MG094
Hepatica transsilvanica
Hepatica acutiloba
Hepatica acutiloba MG084
Hepatica americana
Hepatica americana MG089
Anemone taipaiensis MG016
Anemone zephya MG100
Anemone demissa MG115
Anemone imbricata MG114
Anemone shikokiana MG014
Anemone narcissiflora subsp crinita MG010
Anemone coelestina MG004
Anemone subpinnata MG015
Anemone trullifolia MG101
Anemone dichotoma MG008
Anemone baicalensis MG003
Pulsatilla cernua
Pulsatilla cernua MG040
Pulsatilla chinensis MG041
Pulsatilla multifida MG045
Pulsatilla chinensis F0170
Pulsatilla chinensis F0390
Pulsatilla chinensis MG042
Pulsatilla dahurica MG043
Pulsatilla multifida MG099
Pulsatilla occidemtalis MG097
Pulsatilla patens MG098
Anemone rivularis MG006
Anemone rivularis MG009
Anemone rivularis MG005
Anemone rivularis MG007
Anemone rivularis MG011
Anemone rivularis MG012
Anemone vitifolia F0892
Anemone howellii MG102
Batrachium bungei F0530
Ranunculus sceleratus Z4411
Ranunculus franchetii MG050
Ranunculus ternatus MG061
Halerpestes tricuspis F0690
Ranunculus japonicus Z4443
Caltha scaposa MG118
Adonis amurensis MG001
Adonis davidii MG002
Clematis s.l.
Anemoclema
Hepatica
Anomene II
Pulsatilla
Anemone I

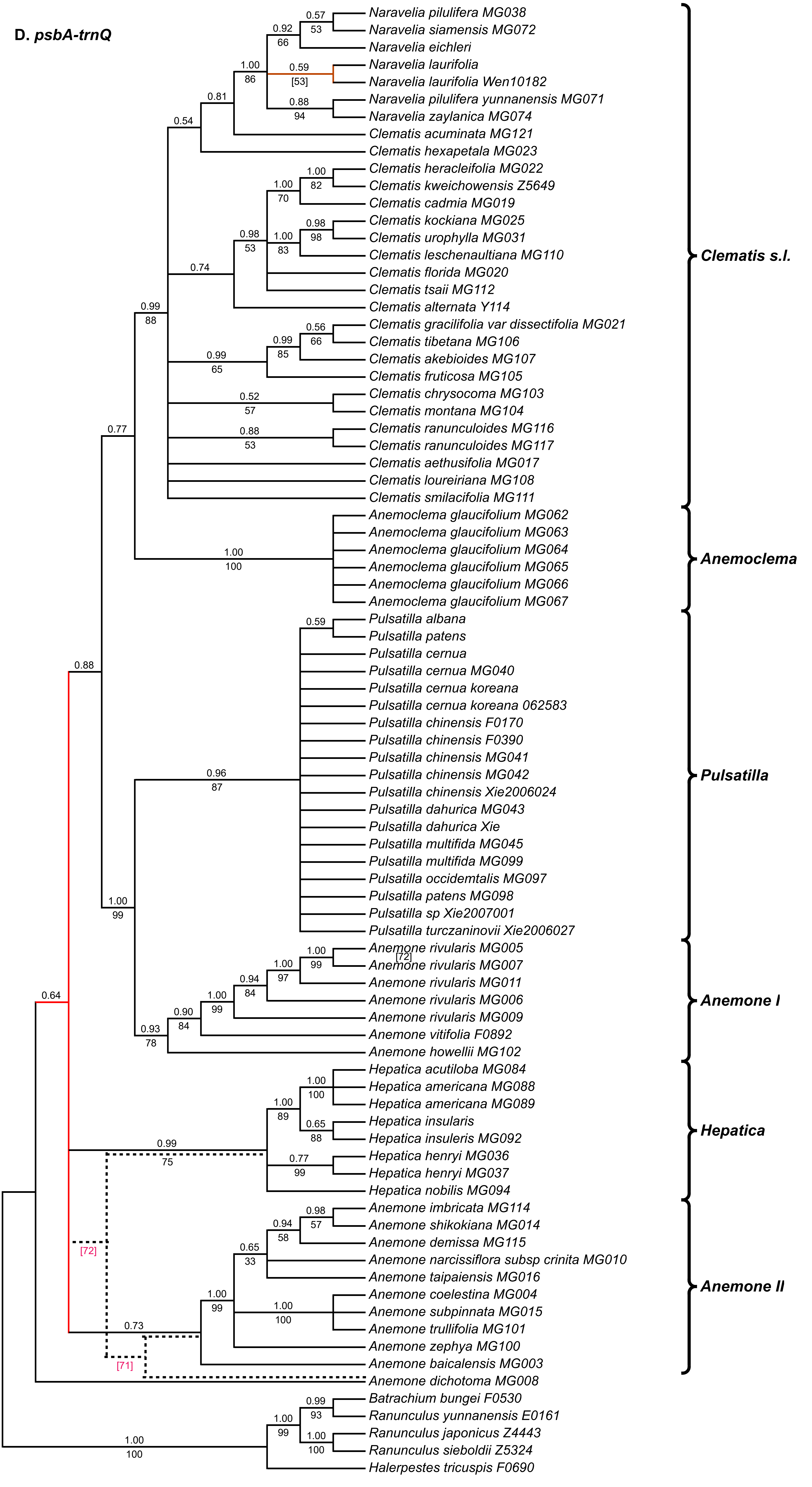

D. psbA-trnQ
Naravelia pilulifera MG038
Naravelia siamensis MG072
Naravelia eichleri
Naravelia laurifolia
Naravelia laurifolia Wen10182
Naravelia pilulifera yunnanensis MG071
Naravelia zaylanica MG074
Clematis acuminata MG121
Clematis hexapetala MG023
Clematis heracleifolia MG022
Clematis kweichowensis Z5649
Clematis cadmia MG019
Clematis kockiana MG025
Clematis urophylla MG031
Clematis leschenaultiana MG110
Clematis florida MG020
Clematis tsaii MG112
Clematis alternata Y114
Clematis gracilifolia var dissectifolia MG021
Clematis tibetana MG106
Clematis akebioides MG107
Clematis fruticosa MG105
Clematis chrysocoma MG103
Clematis montana MG104
Clematis ranunculoides MG116
Clematis ranunculoides MG117
Clematis aethusifolia MG017
Clematis loureiriana MG108
Clematis smilacifolia MG111
Anemoclema glaucifolium MG062
Anemoclema glaucifolium MG063
Anemoclema glaucifolium MG064
Anemoclema glaucifolium MG065
Anemoclema glaucifolium MG066
Anemoclema glaucifolium MG067
Pulsatilla albana
Pulsatilla patens
Pulsatilla cernua
Pulsatilla cernua MG040
Pulsatilla cernua koreana
Pulsatilla cernua koreana 062583
Pulsatilla chinensis F0170
Pulsatilla chinensis F0390
Pulsatilla chinensis MG041
Pulsatilla chinensis MG042
Pulsatilla chinensis Xie2006024
Pulsatilla dahurica MG043
Pulsatilla dahurica Xie
Pulsatilla multifida MG045
Pulsatilla multifida MG099
Pulsatilla occidemtalis MG097
Pulsatilla patens MG098
Pulsatilla sp Xie2007001
Pulsatilla turczaninovii Xie2006027
Anemone rivularis MG005
Anemone rivularis MG007
Anemone rivularis MG011
Anemone rivularis MG006
Anemone rivularis MG009
Anemone vitifolia F0892
Anemone howellii MG102
Hepatica acutiloba MG084
Hepatica americana MG088
Hepatica americana MG089
Hepatica insularis
Hepatica insuleris MG092
Hepatica henryi MG036
Hepatica henryi MG037
Hepatica nobilis MG094
Anemone imbricata MG114
Anemone shikokiana MG014
Anemone demissa MG115
Anemone narcissiflora subsp crinita MG010
Anemone taipaiensis MG016
Anemone coelestina MG004
Anemone subpinnata MG015
Anemone trullifolia MG101
Anemone zephya MG100
Anemone baicalensis MG003
Anemone dichotoma MG008
Batrachium bungei F0530
Ranunculus yunnanensis E0161
Ranunculus japonicus Z4443
Ranunculus sieboldii Z5324
Halerpestes tricuspis F0690
Clematis s.l.
Anemoclema
Pulsatilla
Anemone I
Hepatica
Anemone II
[72]
[71]
[53]

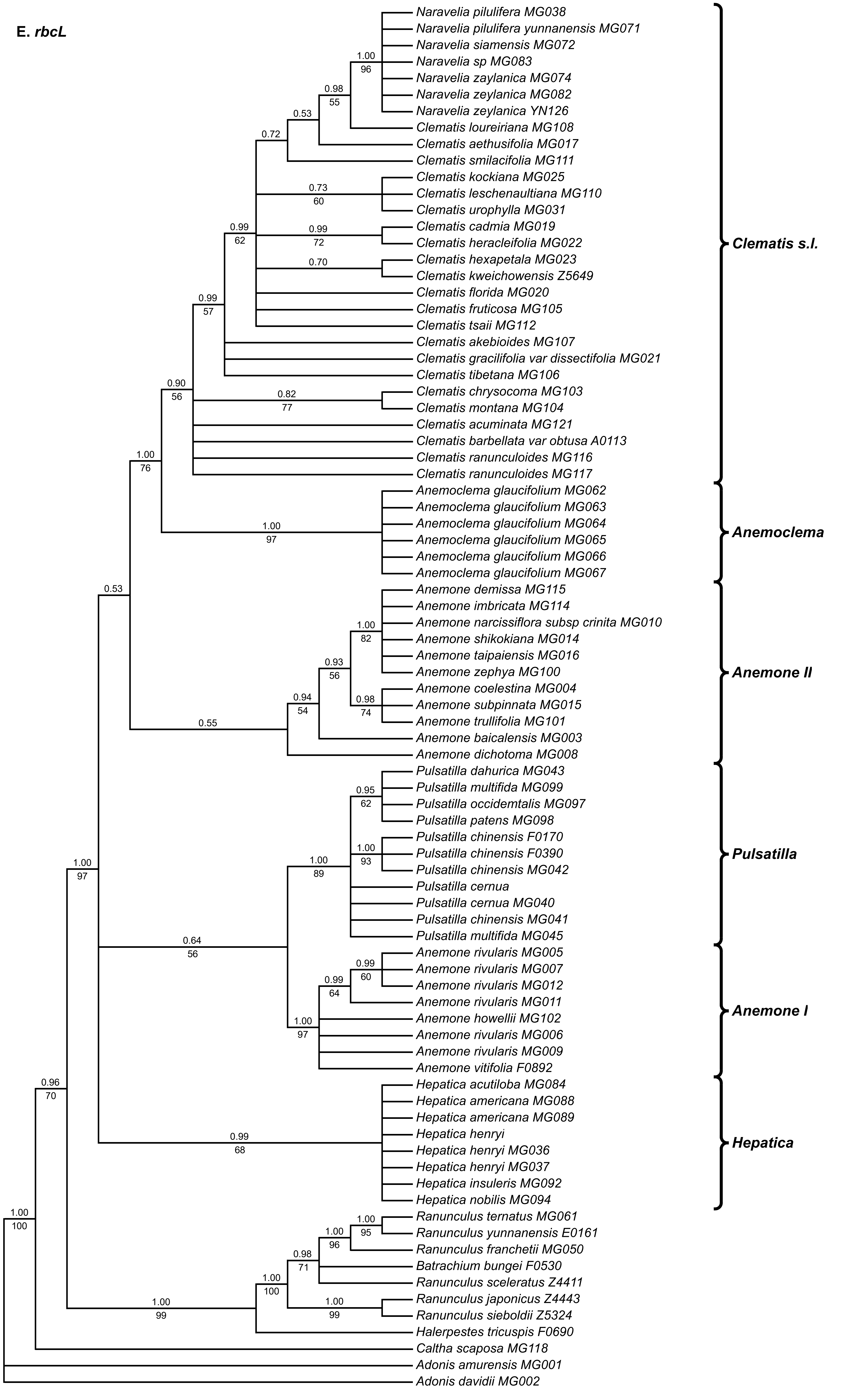

E. rbcL
Naravelia pilulifera MG038
Naravelia pilulifera yunnanensis MG071
Naravelia siamensis MG072
Naravelia sp MG083
Naravelia zaylanica MG074
Naravelia zeylanica MG082
Naravelia zeylanica YN126
Clematis loureiriana MG108
Clematis aethusifolia MG017
Clematis smilacifolia MG111
Clematis kockiana MG025
Clematis leschenaultiana MG110
Clematis urophylla MG031
Clematis cadmia MG019
Clematis heracleifolia MG022
Clematis hexapetala MG023
Clematis kweichowensis Z5649
Clematis florida MG020
Clematis fruticosa MG105
Clematis tsaii MG112
Clematis akebioides MG107
Clematis gracilifolia var dissectifolia MG021
Clematis tibetana MG106
Clematis chrysocoma MG103
Clematis montana MG104
Clematis acuminata MG121
Clematis barbellata var obtusa A0113
Clematis ranunculoides MG116
Clematis ranunculoides MG117
Anemoclema glaucifolium MG062
Anemoclema glaucifolium MG063
Anemoclema glaucifolium MG064
Anemoclema glaucifolium MG065
Anemoclema glaucifolium MG066
Anemoclema glaucifolium MG067
Anemone demissa MG115
Anemone imbricata MG114
Anemone narcissiflora subsp crinita MG010
Anemone shikokiana MG014
Anemone taipaiensis MG016
Anemone zephya MG100
Anemone coelestina MG004
Anemone subpinnata MG015
Anemone trullifolia MG101
Anemone baicalensis MG003
Anemone dichotoma MG008
Pulsatilla dahurica MG043
Pulsatilla multifida MG099
Pulsatilla occidemtalis MG097
Pulsatilla patens MG098
Pulsatilla chinensis F0170
Pulsatilla chinensis F0390
Pulsatilla chinensis MG042
Pulsatilla cernua
Pulsatilla cernua MG040
Pulsatilla chinensis MG041
Pulsatilla multifida MG045
Anemone rivularis MG005
Anemone rivularis MG007
Anemone rivularis MG012
Anemone rivularis MG011
Anemone howellii MG102
Anemone rivularis MG006
Anemone rivularis MG009
Anemone vitifolia F0892
Hepatica acutiloba MG084
Hepatica americana MG088
Hepatica americana MG089
Hepatica henryi
Hepatica henryi MG036
Hepatica henryi MG037
Hepatica insuleris MG092
Hepatica nobilis MG094
Ranunculus ternatus MG061
Ranunculus yunnanensis E0161
Ranunculus franchetii MG050
Batrachium bungei F0530
Ranunculus sceleratus Z4411
Ranunculus japonicus Z4443
Ranunculus sieboldii Z5324
Halerpestes tricuspis F0690
Caltha scaposa MG118
Adonis amurensis MG001
Adonis davidii MG002
Clematis s.l.
Anemoclema
Anemone II
Pulsatilla
Anemone I
Hepatica

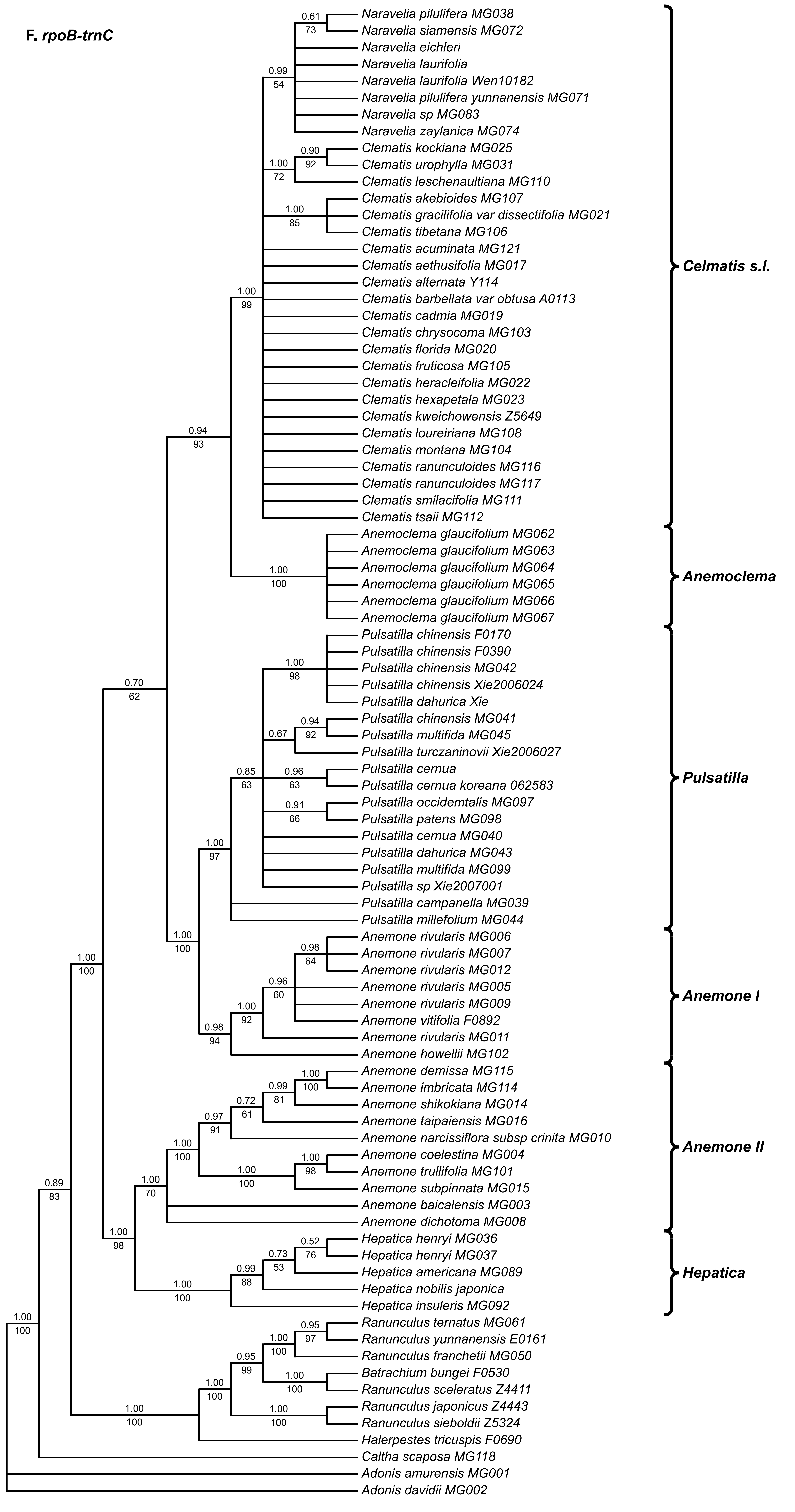
F. rpoB-trnC
Naravelia pilulifera MG038
Naravelia siamensis MG072
Naravelia eichleri
Naravelia laurifolia
Naravelia laurifolia Wen10182
Naravelia pilulifera yunnanensis MG071
Naravelia sp MG083
Naravelia zaylanica MG074
Clematis kockiana MG025
Clematis urophylla MG031
Clematis leschenaultiana MG110
Clematis akebioides MG107
Clematis gracilifolia var dissectifolia MG021
Clematis tibetana MG106
Clematis acuminata MG121
Clematis aethusifolia MG017
Clematis alternata Y114
Clematis barbellata var obtusa A0113
Clematis cadmia MG019
Clematis chrysocoma MG103
Clematis florida MG020
Clematis fruticosa MG105
Clematis heracleifolia MG022
Clematis hexapetala MG023
Clematis kweichowensis Z5649
Clematis loureiriana MG108
Clematis montana MG104
Clematis ranunculoides MG116
Clematis ranunculoides MG117
Clematis smilacifolia MG111
Clematis tsaii MG112
Celmatis s.l.
Anemoclema glaucifolium MG062
Anemoclema glaucifolium MG063
Anemoclema glaucifolium MG064
Anemoclema glaucifolium MG065
Anemoclema glaucifolium MG066
Anemoclema glaucifolium MG067
Anemoclema
Pulsatilla chinensis F0170
Pulsatilla chinensis F0390
Pulsatilla chinensis MG042
Pulsatilla chinensis Xie2006024
Pulsatilla dahurica Xie
Pulsatilla chinensis MG041
Pulsatilla multifida MG045
Pulsatilla turczaninovii Xie2006027
Pulsatilla cernua
Pulsatilla cernua koreana 062583
Pulsatilla occidemtalis MG097
Pulsatilla patens MG098
Pulsatilla cernua MG040
Pulsatilla dahurica MG043
Pulsatilla multifida MG099
Pulsatilla sp Xie2007001
Pulsatilla campanella MG039
Pulsatilla millefolium MG044
Pulsatilla
Anemone rivularis MG006
Anemone rivularis MG007
Anemone rivularis MG012
Anemone rivularis MG005
Anemone rivularis MG009
Anemone vitifolia F0892
Anemone rivularis MG011
Anemone howellii MG102
Anemone I
Anemone demissa MG115
Anemone imbricata MG114
Anemone shikokiana MG014
Anemone taipaiensis MG016
Anemone narcissiflora subsp crinita MG010
Anemone coelestina MG004
Anemone trullifolia MG101
Anemone subpinnata MG015
Anemone baicalensis MG003
Anemone dichotoma MG008
Anemone II
Hepatica henryi MG036
Hepatica henryi MG037
Hepatica americana MG089
Hepatica nobilis japonica
Hepatica insuleris MG092
Hepatica
Ranunculus ternatus MG061
Ranunculus yunnanensis E0161
Ranunculus franchetii MG050
Batrachium bungei F0530
Ranunculus sceleratus Z4411
Ranunculus japonicus Z4443
Ranunculus sieboldii Z5324
Halerpestes tricuspis F0690
Caltha scaposa MG118
Adonis amurensis MG001
Adonis davidii MG002

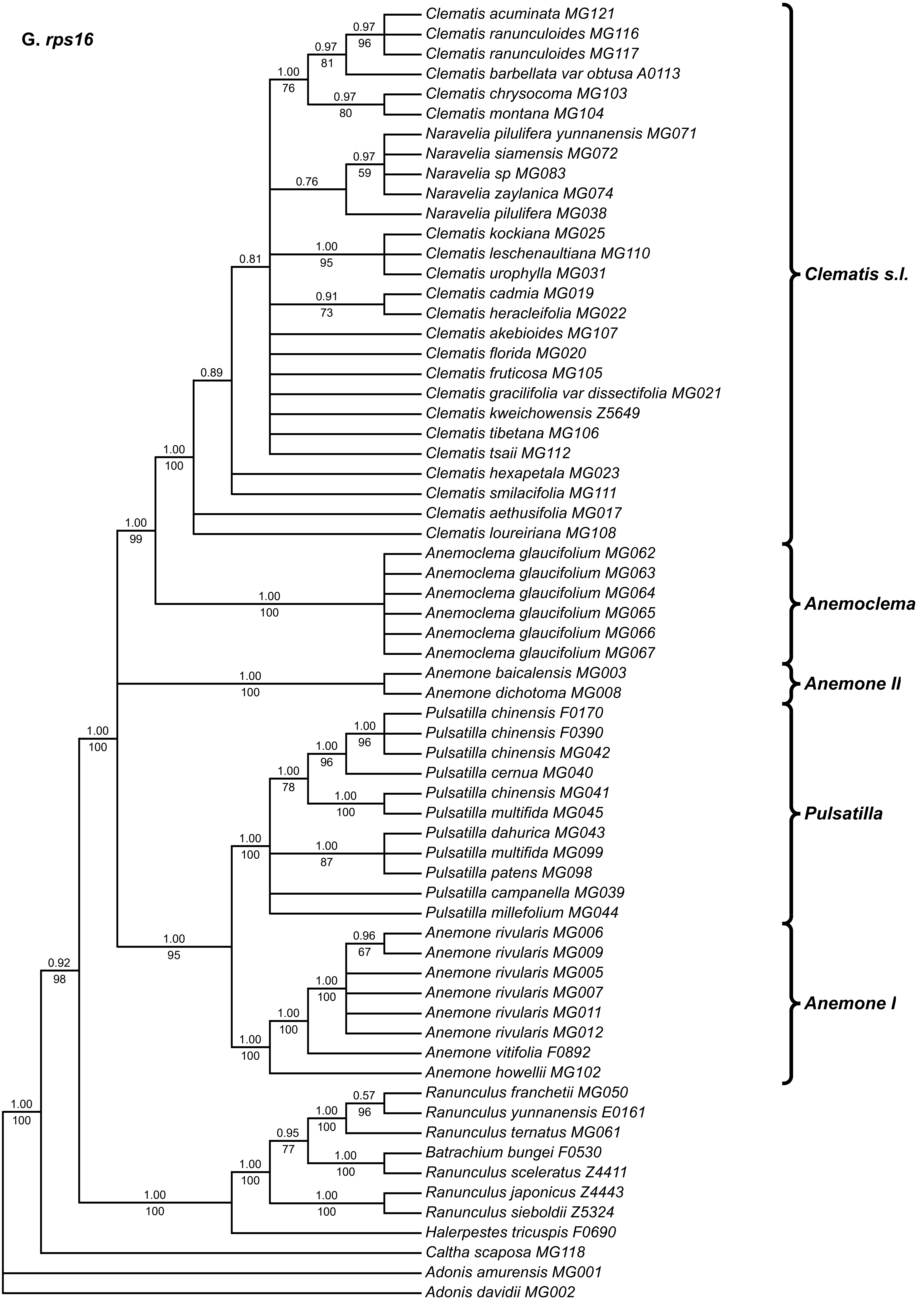
G. rps16
Clematis acuminata MG121
Clematis ranunculoides MG116
Clematis ranunculoides MG117
Clematis barbellata var obtusa A0113
Clematis chrysocoma MG103
Clematis montana MG104
Naravelia pilulifera yunnanensis MG071
Naravelia siamensis MG072
Naravelia sp MG083
Naravelia zaylanica MG074
Naravelia pilulifera MG038
Clematis kockiana MG025
Clematis leschenaultiana MG110
Clematis urophylla MG031
Clematis cadmia MG019
Clematis heracleifolia MG022
Clematis akebioides MG107
Clematis florida MG020
Clematis fruticosa MG105
Clematis gracilifolia var dissectifolia MG021
Clematis kweichowensis Z5649
Clematis tibetana MG106
Clematis tsaii MG112
Clematis hexapetala MG023
Clematis smilacifolia MG111
Clematis aethusifolia MG017
Clematis loureiriana MG108
Anemoclema glaucifolium MG062
Anemoclema glaucifolium MG063
Anemoclema glaucifolium MG064
Anemoclema glaucifolium MG065
Anemoclema glaucifolium MG066
Anemoclema glaucifolium MG067
Anemone baicalensis MG003
Anemone dichotoma MG008
Pulsatilla chinensis F0170
Pulsatilla chinensis F0390
Pulsatilla chinensis MG042
Pulsatilla cernua MG040
Pulsatilla chinensis MG041
Pulsatilla multifida MG045
Pulsatilla dahurica MG043
Pulsatilla multifida MG099
Pulsatilla patens MG098
Pulsatilla campanella MG039
Pulsatilla millefolium MG044
Anemone rivularis MG006
Anemone rivularis MG009
Anemone rivularis MG005
Anemone rivularis MG007
Anemone rivularis MG011
Anemone rivularis MG012
Anemone vitifolia F0892
Anemone howellii MG102
Ranunculus franchetii MG050
Ranunculus yunnanensis E0161
Ranunculus ternatus MG061
Batrachium bungei F0530
Ranunculus sceleratus Z4411
Ranunculus japonicus Z4443
Ranunculus sieboldii Z5324
Halerpestes tricuspis F0690
Caltha scaposa MG118
Adonis amurensis MG001
Adonis davidii MG002
Clematis s.l.
Anemoclema
Anemone II
Pulsatilla
Anemone I
