# Supplementary figures and images for: Phylogenetic reassessment of tribe Anemoneae (Ranunculaceae): Non-monophyly of Anemone s.l. revealed by plastid datasets
Source: PLoS One. 2017 Mar 31;12(3):e0174792. doi: 10.1371/journal.pone.0174792 (PMC5376084; doi:10.1371/journal.pone.0174792)

A. ML tree

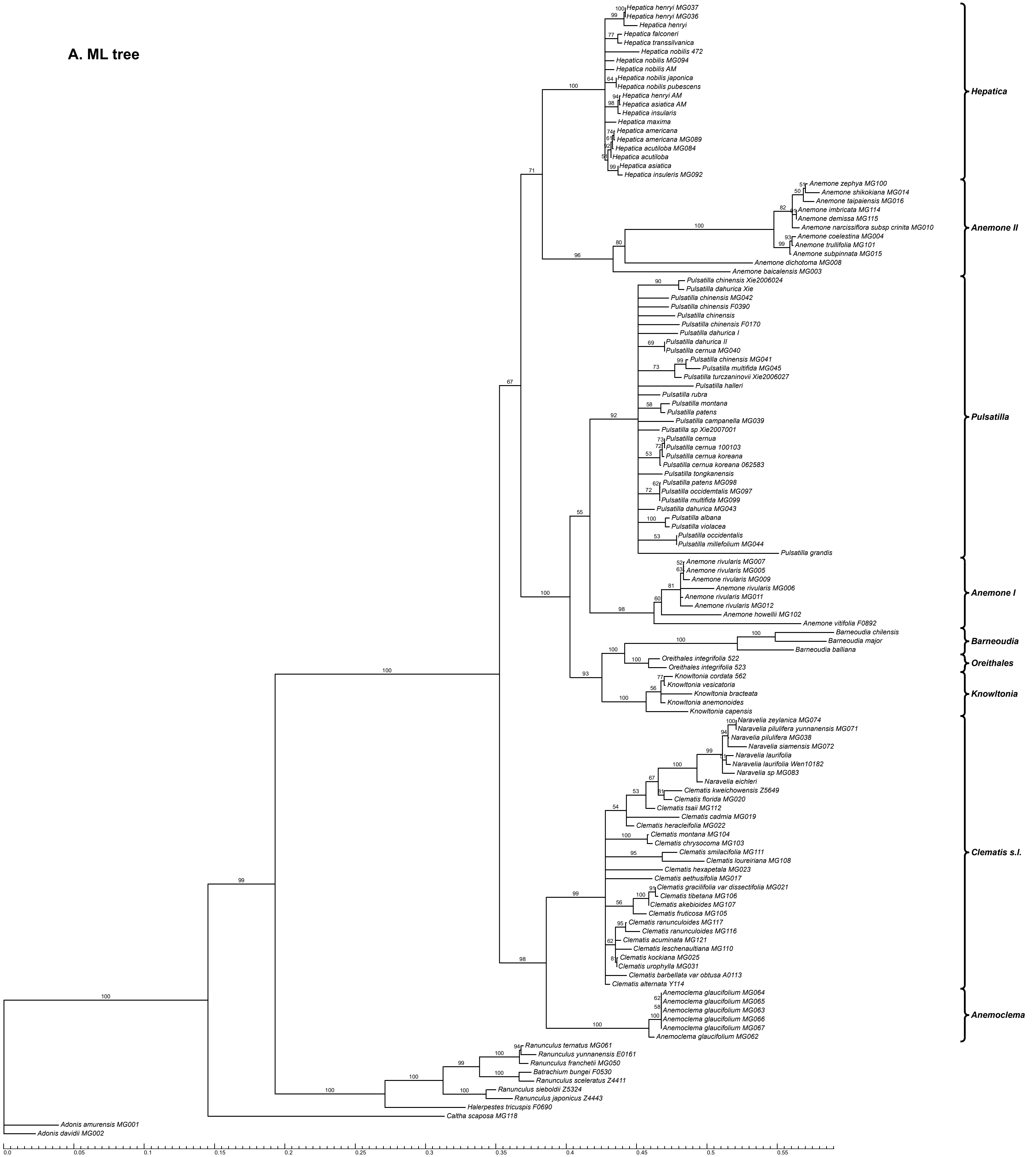

B. BI tree

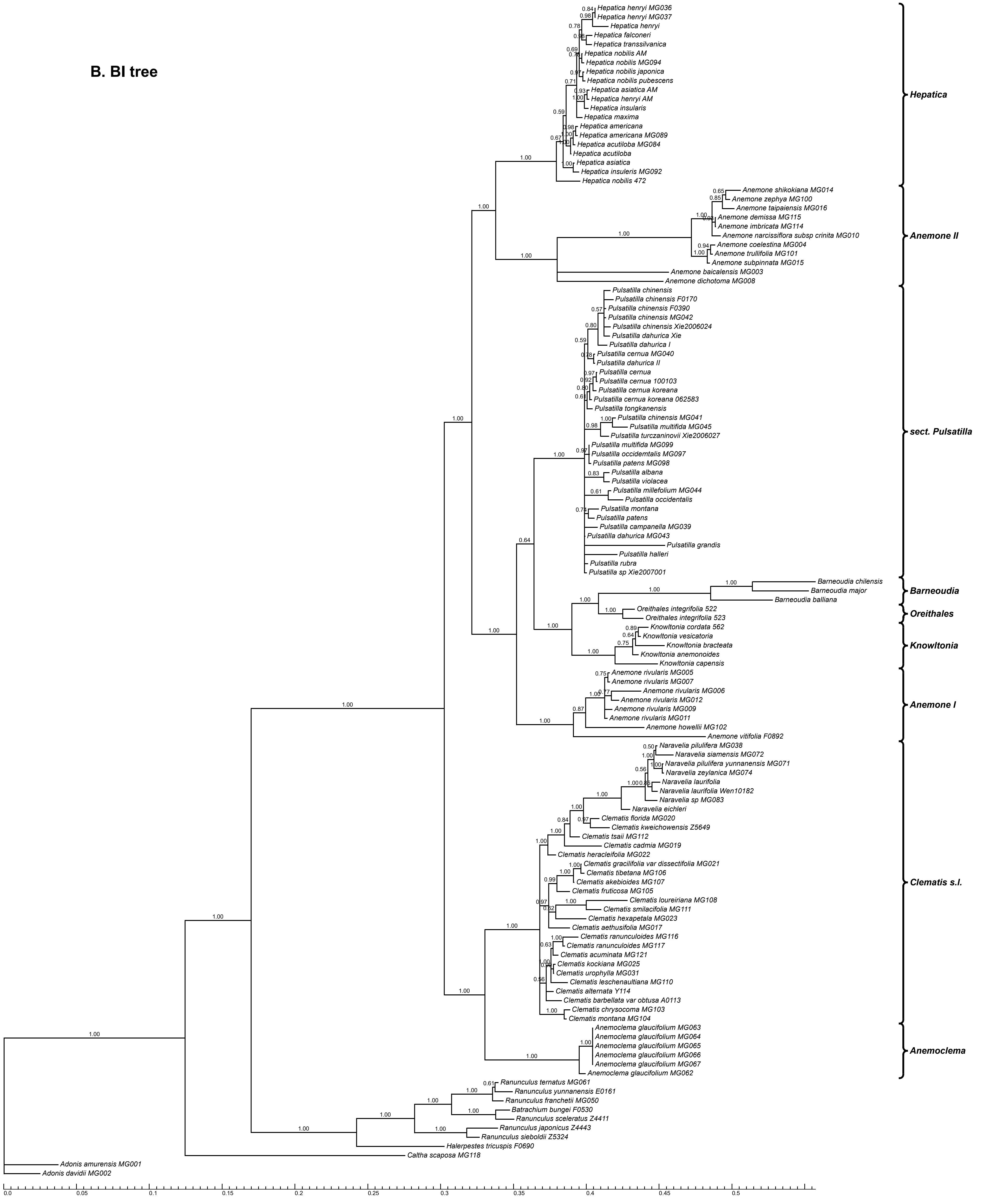

Supplement: S2 Fig — (PDF) [file pone.0174792.s005.pdf]

A. nrITS + *atpB-rbcL*

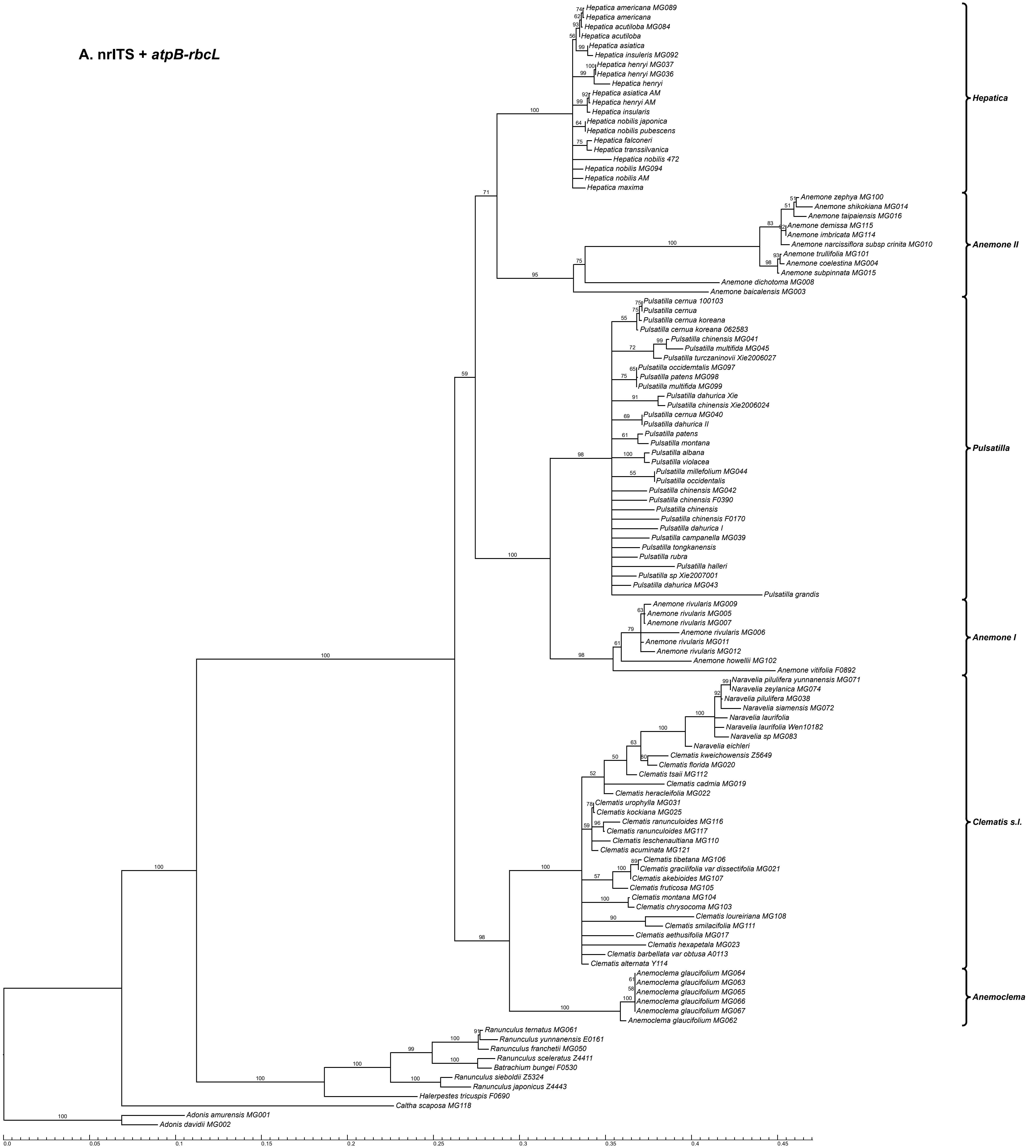

B. Six-plastid-gene

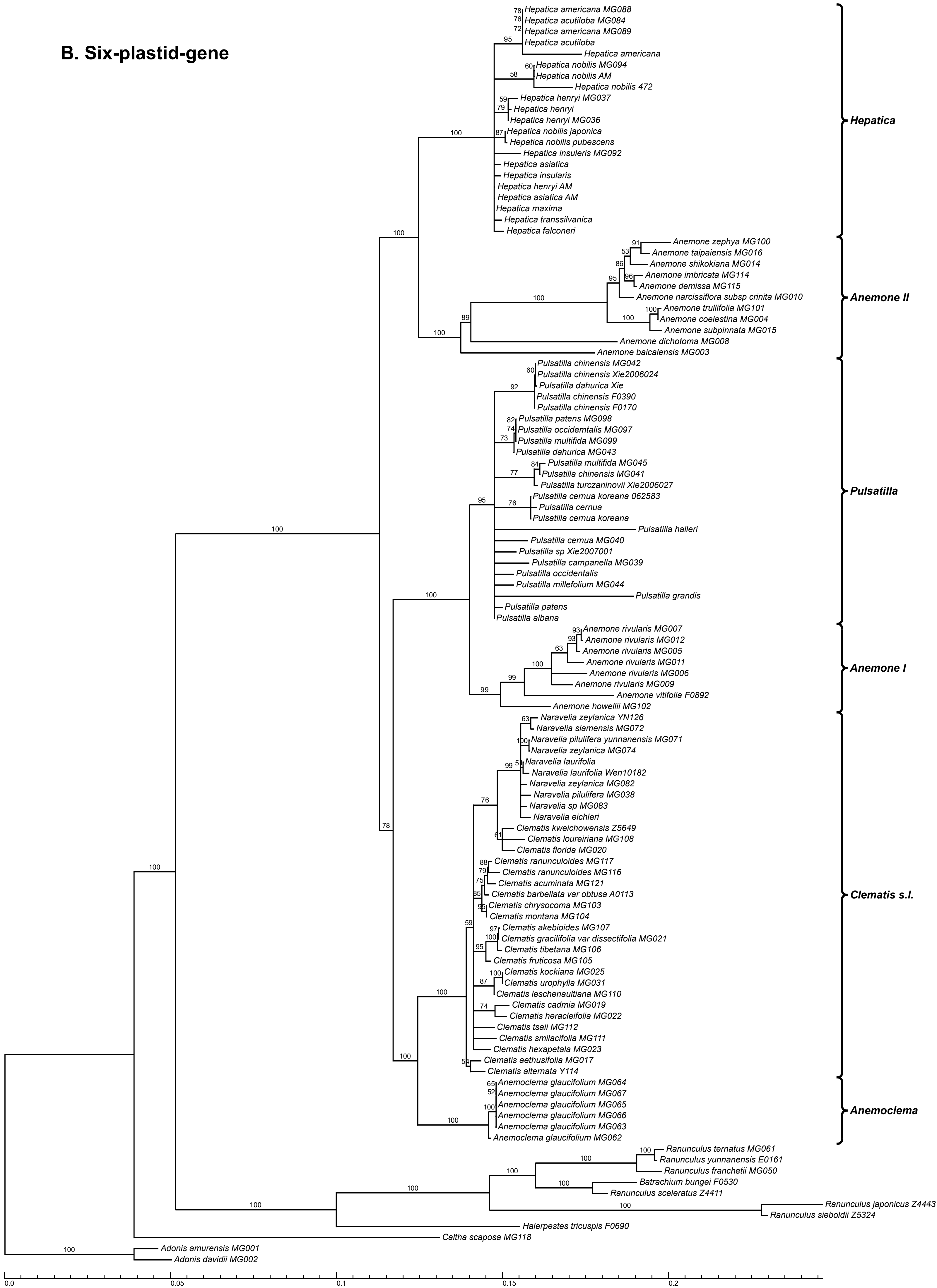

Supplement: S3 Fig — (PDF) [file pone.0174792.s006.pdf]

A. ML tree

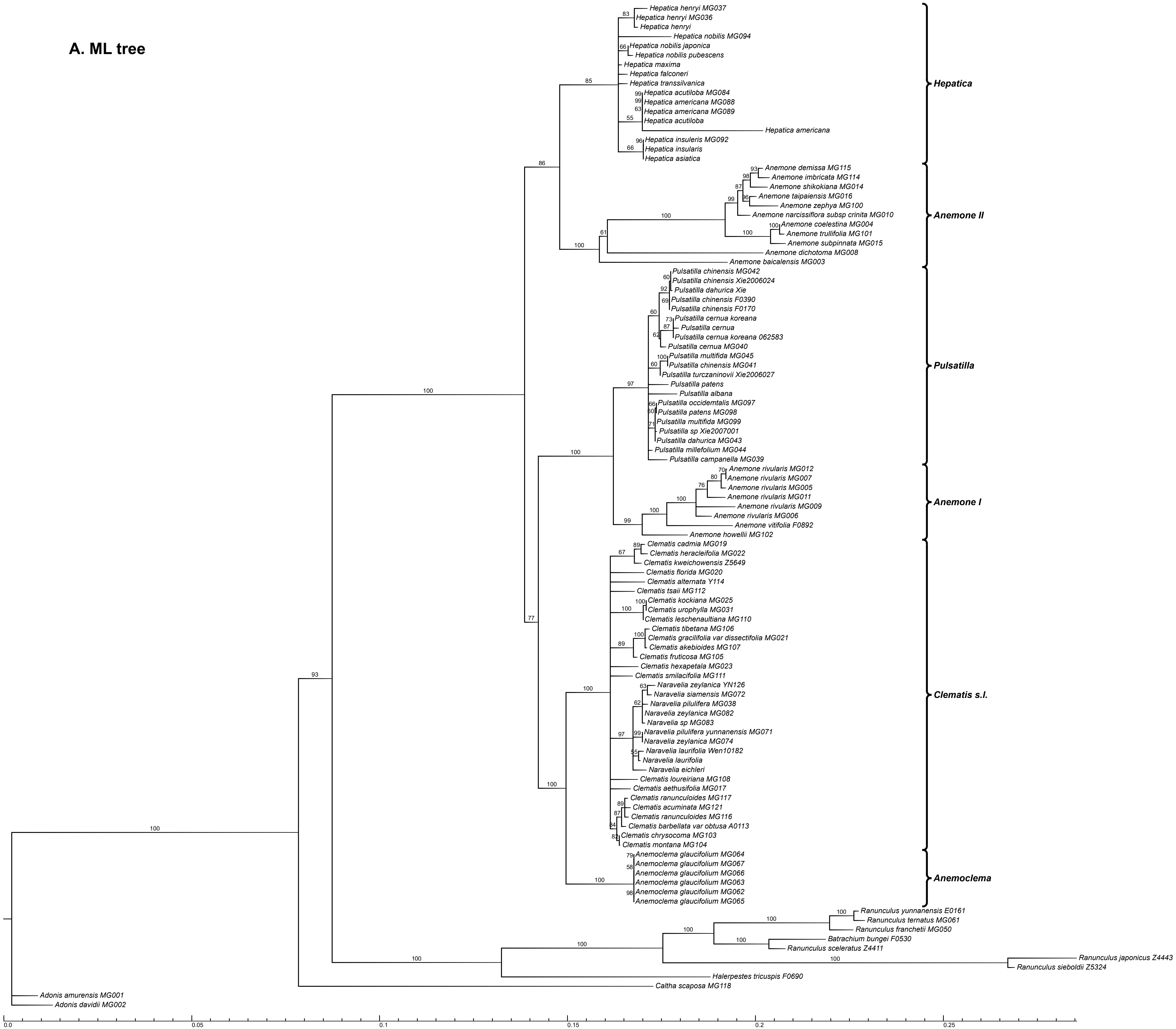

B. BI tree

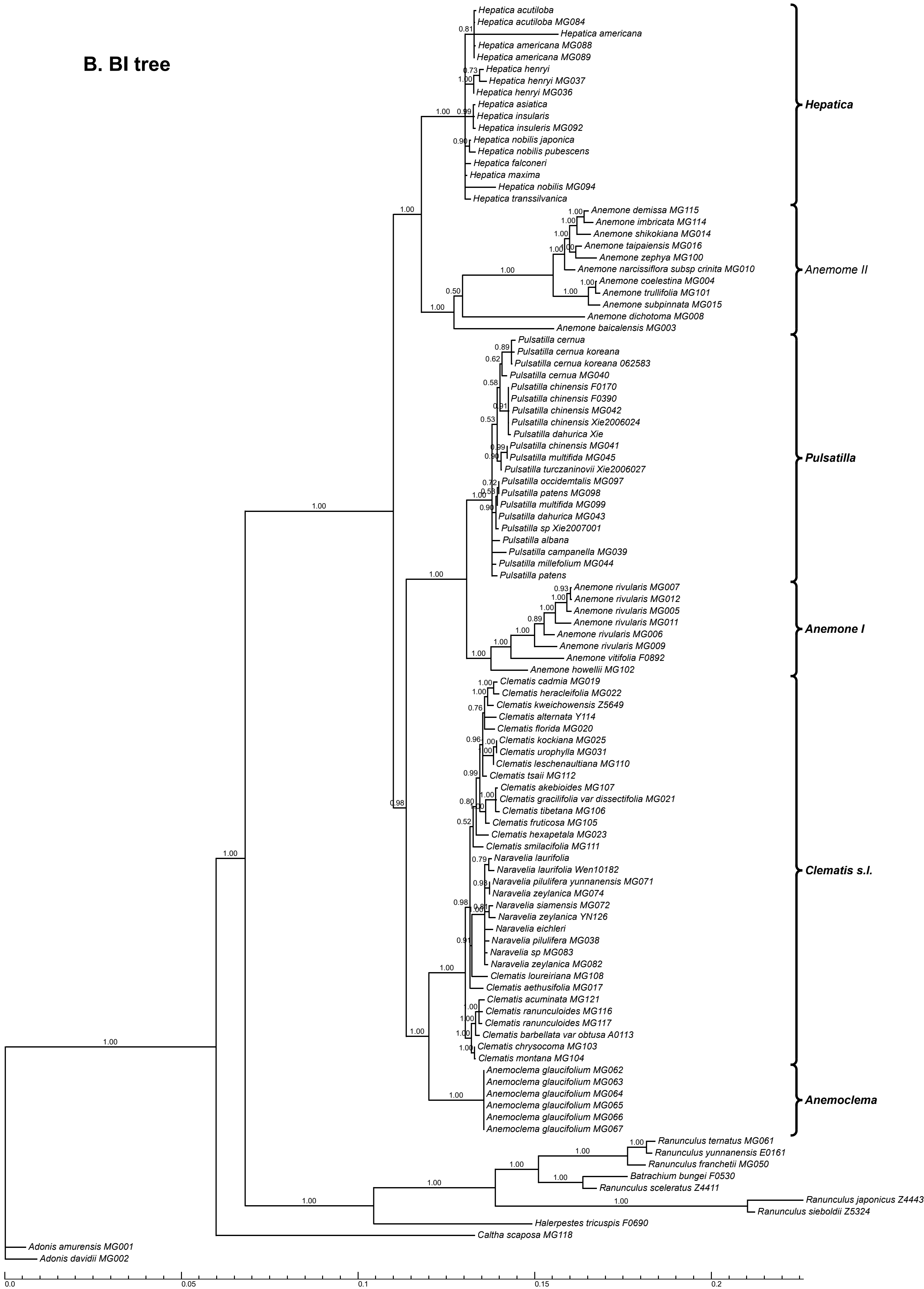

Supplement: S4 Fig — (PDF) [file pone.0174792.s007.pdf]

A. ML tree

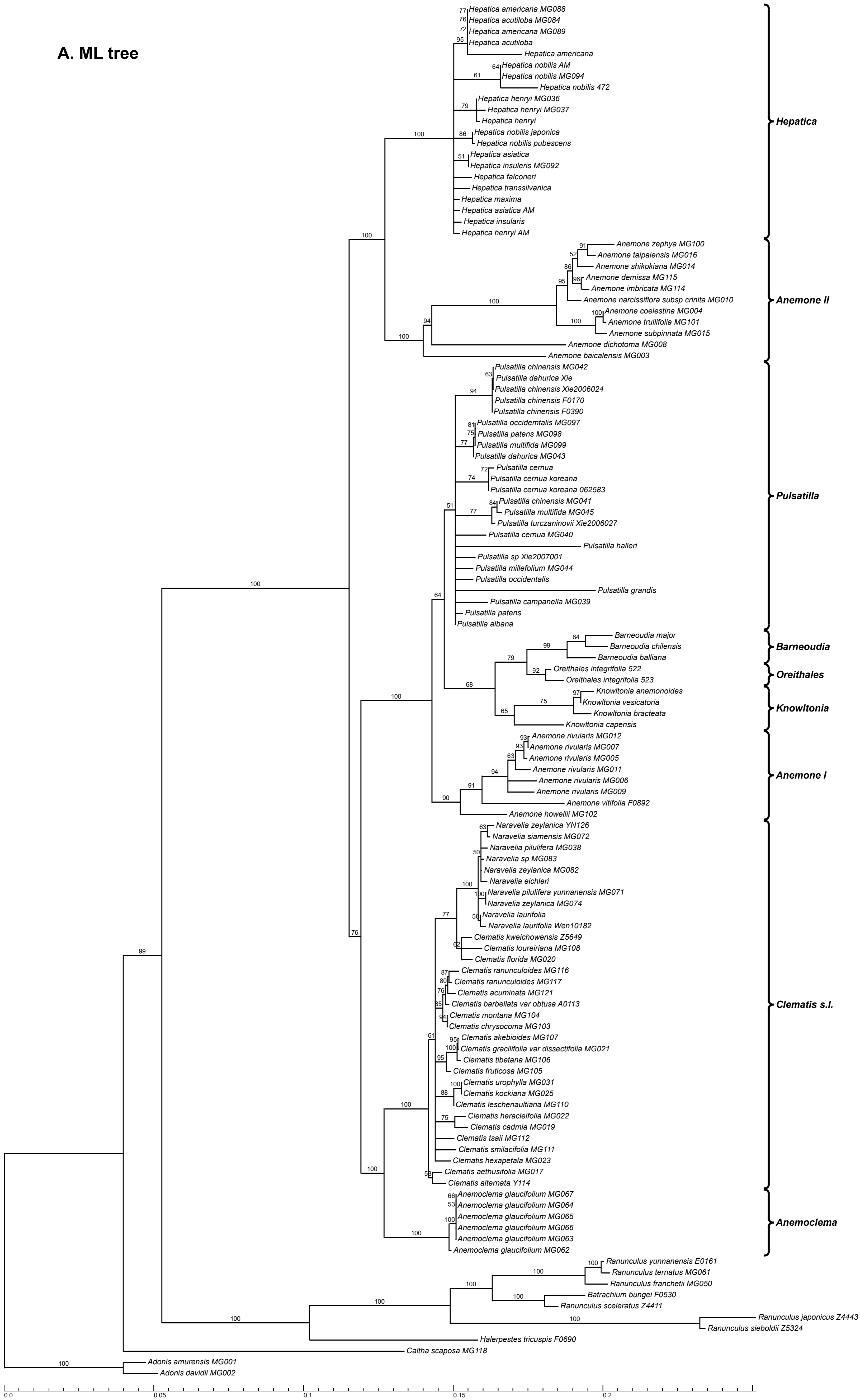

B. BI tree

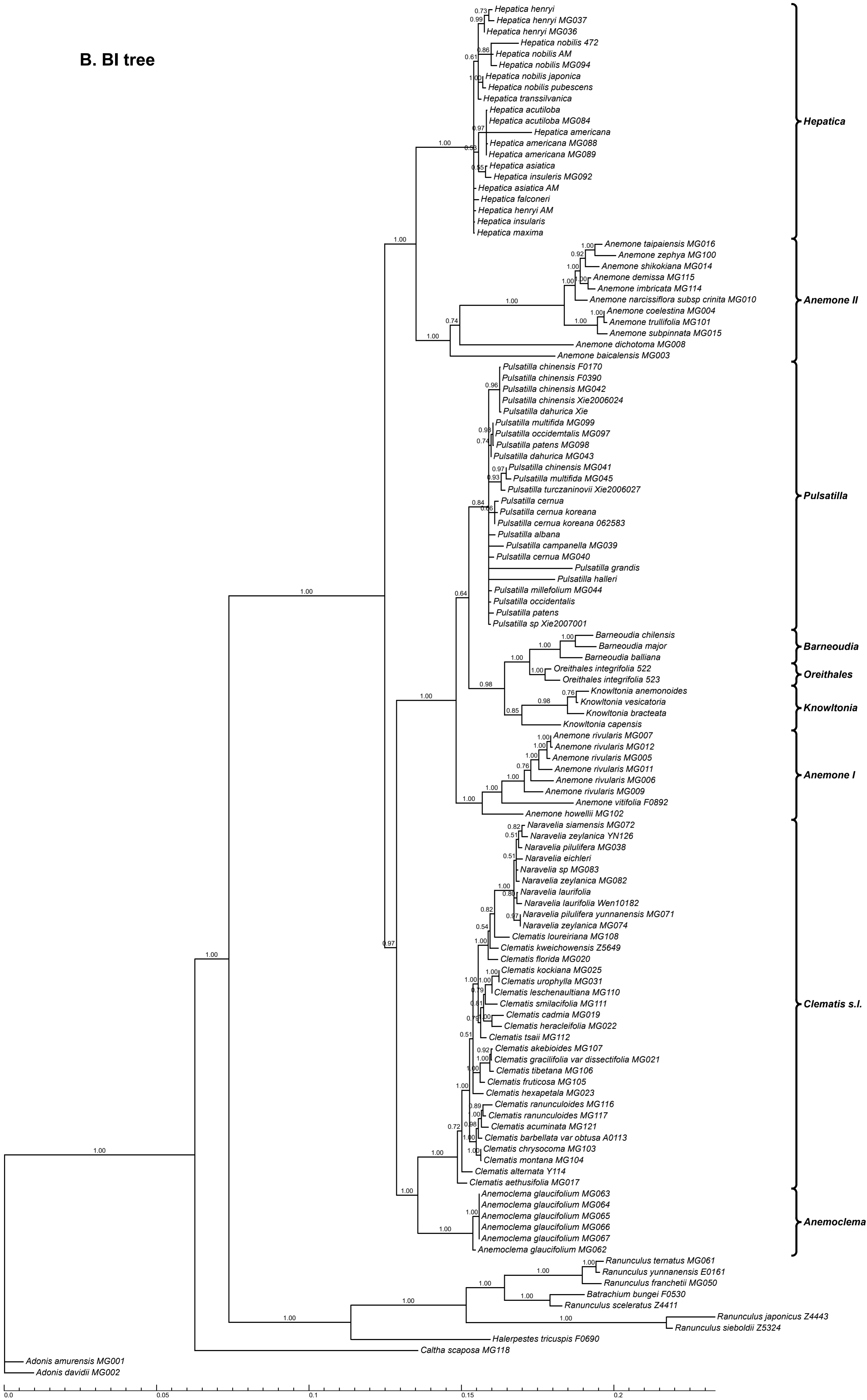

Supplement: S5 Fig — (PDF) [file pone.0174792.s008.pdf]
